# Dysregulation of saliva and fecal microbiota as novel biomarkers of colorectal cancer

**DOI:** 10.3389/fonc.2024.1498328

**Published:** 2024-12-18

**Authors:** Jiamei Rong, Xiaocui Chen, Zhangqin Li, Bona Li, Yang Sun, Yinglei Miao

**Affiliations:** ^1^ Yan’an Hospital Affiliated To Kunming Medical University, Kunming, Yunnan, China; ^2^ Affiliated Hospital of Panzhihua University, Panzhihua, Sichuan, China; ^3^ Department of Gastroenterology, First Affiliated Hospital of Kunming Medical University, Kunming, China

**Keywords:** colorectal cancer, 16s sequencing, machine learning, saliva, feces, differential diagnosis

## Abstract

The aim of this study was to investigate the biomarkers of salivary and fecal microbiota in Colorectal cancer (CRC). Initially, the study scrutinized the microbial community composition disparities among groups. Utilizing Lasso analysis, it sifted through operational taxonomic units (OTUs) to pinpoint distinctive features. Subsequently, by intersecting feature OTUs across groups, it curated a set of core-shared OTUs and devised a corresponding network. Concluding with functional enrichment analysis, the research delved into the divergent biological functions of these microbial communities within the studied groups. Analysis revealed higher bacterial diversity in saliva compared to feces, with distinct differences at both phylum and genus levels. Feces primarily contained Firmicutes, while saliva was dominated by Bacteroidetes and Proteobacteria. Notably, Escherichia-Shigella and Fusobacterium in feces and Streptococcus in saliva showed increasing abundance from average to adenoma to colorectal cancer. Specific dominant flora was identified within and between groups, including CRC and adenomas across different stages. Seventeen core shared OTUs were identified, and networks of shared OTUs were constructed for each group. Functional enrichment analysis highlighted distinct microbial community functions among the groups. This study’s findings on characteristic OTUs in saliva and fecal samples offer valuable insights for distinguishing between healthy individuals, adenoma patients, and those with colorectal cancer. This study identified distinctive OTUs in saliva and feces to distinguish between healthy individuals, adenoma patients, and those with CRC, offering a valuable diagnostic reference.

## 1 Introduction

Colorectal cancer (CRC) is one of the three common malignant tumors (1). It is estimated that the global CRC burden will increase by 60% in 2030, with more than 2.2 million new cases and nearly 1.1 million CRC deaths (2). At present, it is generally believed that the occurrence and development of CRC follows the sequence of “normal mucosa-adenoma-carcinoma”, and about 80%-95% of CRC develops from adenomatous polyp, which generally takes 5-10 years or even longer, providing an important time window for early diagnosis and clinical intervention (3). CRC screening methods in our population are not uniform and have their own limitations (4, 5). Therefore, in-depth study of the imbalance of intestinal microecology during the occurrence and development of CRC, and mining for targeted and sensitive biomarkers to monitor early CRC can lay an important theoretical basis and practical scheme for improving early diagnosis and treatment management strategies, which are of great significance for improving the quality of life of patients at high risk of CRC.

More and more evidence shows that chronic inflammation, host genetic susceptibility and environmental factors are related to the progress of CRC (6). Environmental factors are very important to the composition and function of intestinal microorganisms, and changes in them cause alterations in host gene expression, metabolic regulation and local and systemic immune responses, thus affecting the development of cancer (7). In recent years, more and more studies have reported that the composition and diversity of intestinal flora play an important role in the occurrence and development of chronic liver disease, irritable bowel syndrome, inflammatory bowel disease, CRC and other multi-system diseases (8–10). Imbalance of intestinal flora is considered a potentially important cause of CRC (11). Different from the causal role of Helicobacter pylori in gastric cancer, the specific microorganism that causes CRC has not been determined. Therefore, the repeated identification and verification of cancer-causing microorganisms in patients with diseases is of great significance for understanding its role in the pathogenesis of CRC and finding the best treatment for CRC.

The gastrointestinal tract begins in the mouth, and the oral microflora is the second largest microflora after the gastrointestinal tract because of its complex composition. In recent years, it has been found that CRC is not only related to intestinal microflora, but also closely related to in oral microflora (12, 13). However, few studies pay attention to the differences of oral microflora between adenoma and CRC patients and healthy people. There is evidence that there may be an “oral-intestinal axis” in the pathogenesis of CRC (14), that poor oral hygiene may cause cancer by changing the number of specific oral bacteria, and that periodontal disease may increase systemic inflammation, leading to immune disorders and changes in the intestinal flora, which may affect the occurrence of CRC (15). In this topic, we explore whether there is a potential microbial basis between oral microorganisms and CRC, and further explore this potential oral-intestinal axis to find new non-invasive biomarkers of CRC.

At present, the role of intestinal microbiota in the occurrence and development of intestinal cancer has become more and more clear, but the details of how oral microbiota changes intestinal microbiota and affects the occurrence of intestinal cancer are still unclear. In this study, 16SrRNA high-throughput sequencing was used to analyze the microbial diversity and species abundance in normal, adenoma and CRC in saliva and feces, with a large sample size and strict exclusion criteria. Linking the two provides a theoretical basis for the early screening and pathogenesis of CRC from the perspective of salivary and intestinal microecology.

## 2 Materials and methods

### 2.1 Patient selection

Patients who underwent colonoscopy in the Digestive Endoscopy Laboratory of the First Affiliated Hospital of Kunming Medical University from October 2020 to September 2022 and were pathologically diagnosed with adenoma and bowel cancer were selected, while healthy volunteers matching the age, gender and body mass index of patients with adenoma and bowel cancer were recruited as healthy control groups. A total of 122 normal samples, 122 adenoma samples and 117 colorectal cancer (CRC) samples were enrolled in this study. Saliva and fecal samples were collected separately for each sample. These samples were classified into 8 groups, specifically: group A: fecal of the normal group, group B: fecal of the adenoma group, group C: fecal of the CRC group, group D: saliva of the normal group, group E: saliva of the adenoma group and group H: saliva of the CRC group. The studies involving human participants were reviewed and approved by [the First Affiliated Hospital of Kunming Medical University]. The patients provided their written informed consent to participate in this study. All the procedures were conducted in accordance with the “Declaration of Helsinki”.

### 2.2 Sample collection

#### 2.2.1 Saliva collection

No brushing of teeth after getting up in the morning and fasting 2 hours before sampling. A sterile, enzyme-free collection tube was left in the mouth for at least 1 min and 2 mL of non-irritating saliva. After collection, freeze in a -80°C freezer within 2 hours.

#### 2.2.2 Fecal collection

Fresh stool was collected and divided into about 50-100 mg into a sterile centrifuge tube and frozen in a -80°C refrigerator within 2 hours.

### 2.3 DNA extraction and 16S sequencing

The CTAB/SDS method was used to extract the total genome DNA in samples. PCR amplification was performed with a diluted genomic DNA template. Following the manufacturer’s recommendations, sequencing libraries were generated with NEBNext^®^Ultra™ IIDNA Library Prep Kit (Cat No. E7645). The library quality was evaluated on the Qubit@ 2.0 Fluorometer (Thermo Scientific) and Agilent Bioanalyzer 2100 system. Finally, the library was sequenced on an Illumina NovaSeq platform and 250 bp paired-end reads were generated.

### 2.4 Operational taxonomic units distribution and alpha diversification analysis

The valid sequence clusters of the samples were grouped into OTUs based on the sequencing data of 16S rRNA of each sample with a similarity threshold of > 98%. OTUs with relative abundance less than 0.01% in the sample were excluded, and the remaining OTUs were utilized for follow-up analysis. Then, an alpha diversity analysis was carried out, and the rarefaction curves and box plots were plotted.

### 2.5 Beta diversity analysis

The PCoA clustering plots based on all samples were utilized to illustrate the differences between the different subgroups in the fecal group and the saliva group. Subsequently, within-group and inter-group similarity analyses were performed separately for the fecal and saliva groups using ANOSIM analysis.

### 2.6 Analysis of species composition diversity

Furthermore, we observed differences in microbial community composition between the six groups at the phylum and genus level. The phylum horizontal bar stack (TOP10) and analysis of differences box-plot was plotted. Meanwhile, genus horizontal bar stack (TOP12) and analysis of differences box-plot was also plotted.

### 2.7 Analysis of flora differences

The Linear Discriminant Analysis (LDA) Effect Size analysis was carried out to find out which OTUs were causing the differences in the communities according to LDA score > 2 and p < 0.05. The analysis was performed between groups A and B, A and C, B and C, D and E, D and H, and E and H. Of these, groups A and B and D and E were analyzed to identify the specific dominant flora associated with adenomas in feces and saliva. The groups A and C and D and H were analyzed to determine the specific dominant groups of bacteria associated with CRC. The groups B and C and E and H were analyzed to determine the specific dominant groups of bacteria associated with CRC and adenomas. Fecal and saliva group samples of adenoma and early CRC were analyzed to identify specific dominant flora associated with adenoma and early CRC. Samples from the fecal and saliva groups of early and advanced CRC were analyzed to identify the specific dominant flora associated with early (stage I-II) and later stages (Stage III-IV) of CRC.

### 2.8 Machine learning filtering feature OTUs

The data of groups A and B, A and C, B and C, D and E, D and H, and E and H were randomly classified into training and validation sets in the ratio of 5:5. The Least Absolute Shrinkage and Selection Operator (LASSO) analysis was performed with the glmnet package (16) to build a diagnostic model and to filter the feature OTUs. The specific groupings were as follows: 244 cases each in groups A and B, and D and E, including 122 cases in the training set (A: 61, B: 61) and 122 cases in the validation set (A: 61, B: 61); 239 cases each in groups A and C, and D and H, including 119 cases in the training set (A: 62, C: 57) and 120 cases in the validation set (A: 60, C: 60); 239 cases each in groups B and C, and E and H, including 119 cases in the training set (A: 62, B: 57) and 120 cases in the validation set (A: 60, B: 60). Then, the AUC value of the receiver operating characteristic (ROC) curve was computed to assess the predictive accuracy of the model. Lastly, POD difference analysis was performed.

### 2.9 Network analysis of shared OUT

The OTUs that were expressed in at least 30% of the 6 groups of samples and had more than 300 reads in a single sample were identified as core-shared OTUs. Afterwards, the Spearman correlation coefficients between the core-shared OUTs were computed via the psych package and the core-shared OUT network was created.

### 2.10 Analysis of flora and clinical features

Pearson correlation coefficients were computed for different clinical features and microbial community genus levels. The top 20 genera with the highest relative abundance were selected for analysis, and the p < 0.05 relationship pairs were presented. Thereafter, statistical analyses of differences were performed for these 20 genera in different clinical subgroups (cancer sites and stage).

### 2.11 Functional enrichment analysis

For exploring the pathways involved in inter-group differences in microbial communities, Kyoto Encyclopedia of Genes and Genomes (KEGG) enrichment analysis was performed via PICRUSt2 (17). The analysis was performed between groups A and B, A and C, B and C, D and E, D and H, and E and H.

### 2.12 Statistical analysis

All bioinformatics analyses were carried out in R language. The data of different groups were compared by Wilcoxon test.

## 3 Results

### 3.1 A total of 1567 common core OTUs were identified for alpha diversification analysis

**Figure 1:**
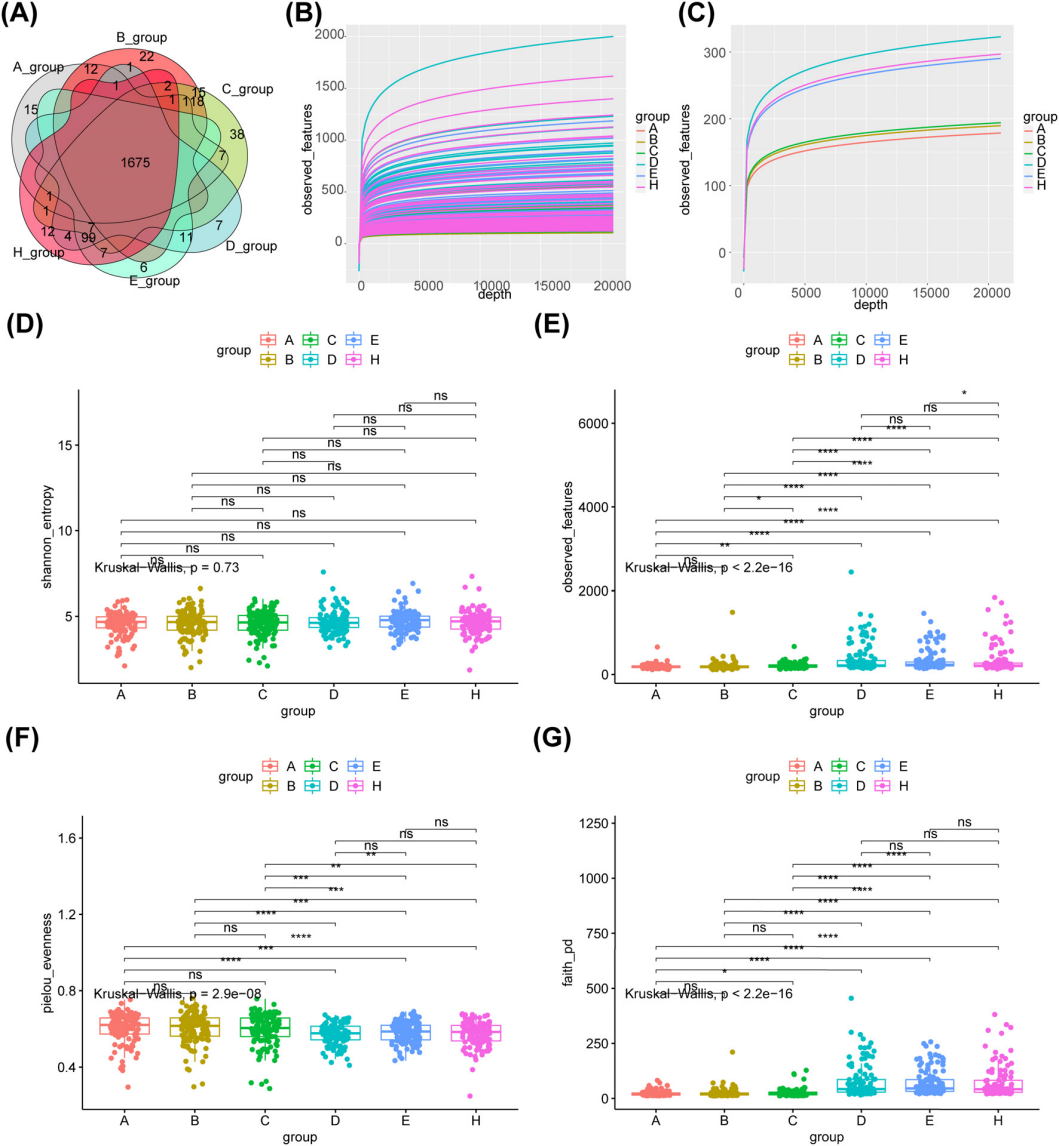
Species abundance and bacterial diversity in six groups. **(A)**Venn diagram shows the common or unique OTUs in different groups of samples. **(B)** The dilution curve of observed_features diversity index shows that the sequencing of the sample is enough to reflect the species diversity in the sample. **(C)** The diversity index of saliva group was greater than that of feces group.α-diversity is indicated by the Shannon **(D)**, observed OTU **(E)**, Evenness **(F)** and PD diversity **(G)** indices. A- healthy group feces, B- adenoma group feces, C- colon cancer group feces, D- healthy group saliva, E- adenoma group saliva, H- colon cancer group saliva, Core- common OTUs. ns, no significance; *: p < 0.05; **: p < 0.01; ***: p < 0.001; ****: p < 0.0001.

A total of 1675 common core OTUs were found in the 6 groups, of which 15 were OTUs specific to group A, 22 to group B, 38 to group C, 7 to group D, 6 to group E, and 12 to group H (**Figure 1A**). The rarefaction curves indicated that the sequencing data were sufficient to reflect the diversity and abundance of species in the samples (**Figure 1B**). And the diversity index of the saliva group was greater than that of the fecal group (**Figure 1C**). Alpha analysis of the indices revealed that the p-values for the observed OTU index, evenness index and PD index were less than 0.05 in the six groups (**Figures 1D–G**). The evenness index was greater in groups B and C than in groups E and F respectively, while the opposite was true for the observed OTU index and PD index (**Figures 1E, F** **)**. The results of the within-group analysis of variance revealed that the observed OTU and PD indices were significantly higher in group C than in group A (**Figures 1E, F** **)**, and that there were significant differences in the observed otu indices between groups B and C (**Figure 1E**). Meanwhile, there was a significant difference in the observed OTU index between group E and group H (**Figure 1E**).

### 3.2 Beta diversity analysis revealed differences between different subgroups of the stool group and the saliva group

**Figure 2:**
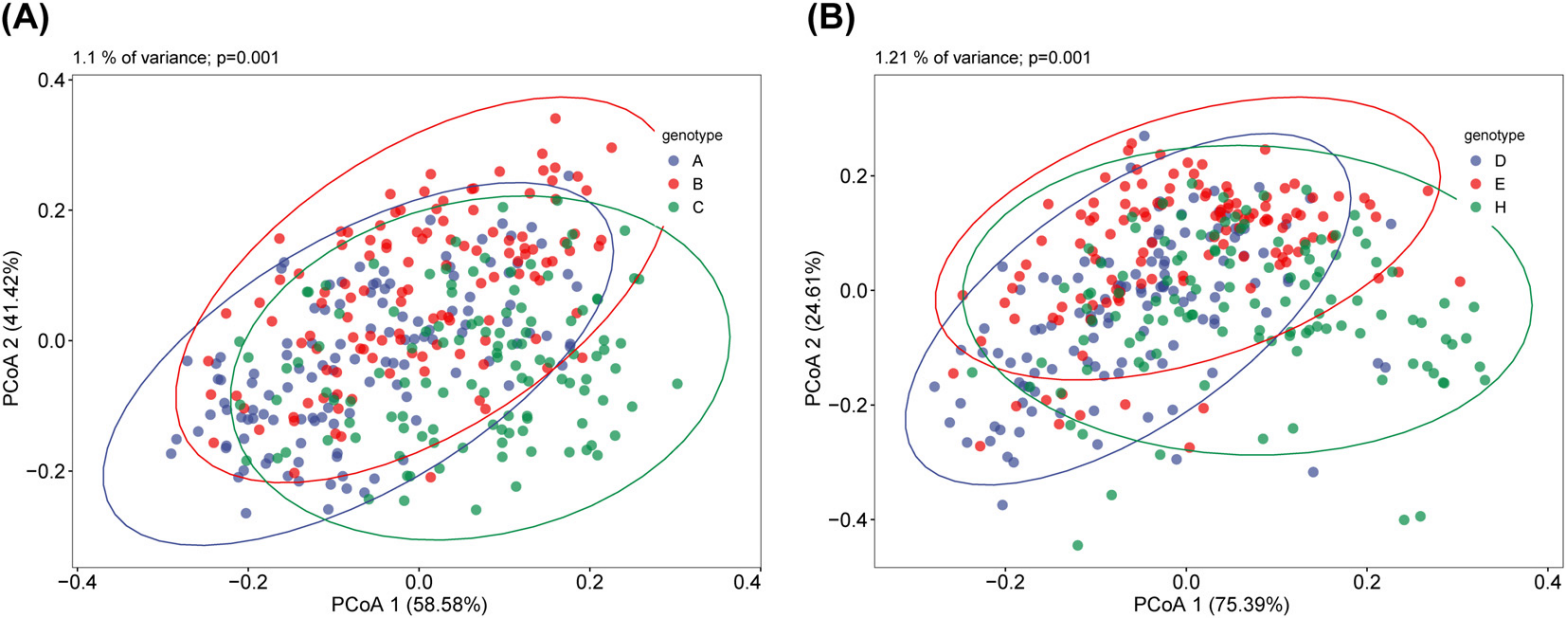
Principal Coordinate Analysis (PCoA) of saliva and fecal microbiota. β diversity can reflect the overall differences between different groups of samples. There are significant differences in β diversity between fecal **(A)** and saliva **(B)** among the three groups. ANOSIM analysis showed that the difference between fecal and saliva groups reached a significant level, and the P values in each group were less than 0.05.

The differences between the different subgroups of the fecal and saliva groups were shown in **Figures 2A, B**. The results of the similarity analysis indicated that there were significant differences within (C VS B, C VS A and A VS B) and between groups in the fecal group (**Table 1**). Similar results were seen in the saliva group (**Table 2**).

**Table 1:** The results of ANOSIM in the fecal group.

Group	R	P-value
A_VS_B	0.011	0.002
A_VS_C	0.014	0.001
B_VS_C	0.01	0.005
ALL	0.015	0.001

R: correlation coefficient. -1<R<0, indicates that the two variables are negatively correlated. 0<R<1, indicates that the two variables are positively correlated. P<0.05, indicates a significant difference.

**Table 2:** The results of ANOSIM in the saliva group.

Group	R	P-value
D_VS_E	0.01	0.007
D_VS_H	0.022	0.001
E_VS_H	0.01	0.004
All	0.019	0.001

R: correlation coefficient. -1<R<0, indicates that the two variables are negatively correlated*.* 0<R<1, indicates that the two variables are positively correlated. *P*<0.05, indicates a significant difference.

### 3.3 Species composition at phylum and genus level was diverse

**Figure 3:**
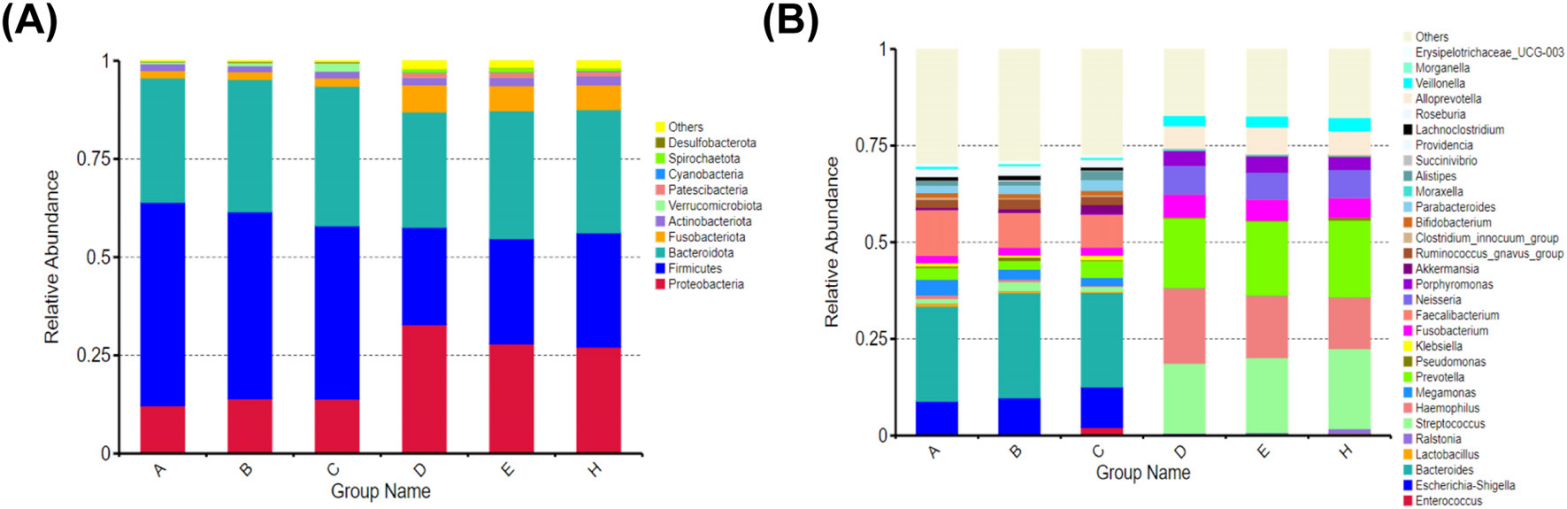
Relative abundance of microbial communities at the phylum and genus levels in six groups. **(A)** There is little difference in species composition between the two groups at the phylum level, but the abundance ratio is different. **(B)** Although there are great differences in the composition of oral and intestinal flora at the genus level, there are common genera.

At the phylum level, the main species in the intestine and mouth were composed of Firmicutes, Bacteroidetes, Proteobacteria, Actinobacteria, and Fusobacteria. In feces, the main species was Firmicutes. Its abundance gradually decreased along the normal-adenoma-bowel cancer, which was negatively correlated with the development of bowel cancer. And in saliva, the main species were Bacteroidetes and Proteobacteria. Proteobacteria abundance gradually decreases along normal-adenoma-bowel cancer (**Figure 3A**). At the genus level, the top five feces were Bacteroides, Faecalibacterium, Escherichia-Shigella, Megamonas, and Prevotella. The top five species in saliva were Hemophilus, Streptococcus, Prevotella, Neisseria, Alloprevotella. However, there were Hemophilus, Streptococcus, Prevotella, Fusobacterium and other fungi in the mouth and intestines. Escherichia-Shigella was less in saliva and feces in the normal group and was enriched in the bowel cancer group. The abundance of Escherichia-Shigella and Fusobacterium in feces gradually increased along the normal-adenoma-bowel cancer, and the abundance of Streptococcus in saliva also followed this rule (**Figure 3B**).

### 3.4 The significant species were identified in different disease groups

**Figure 4:**
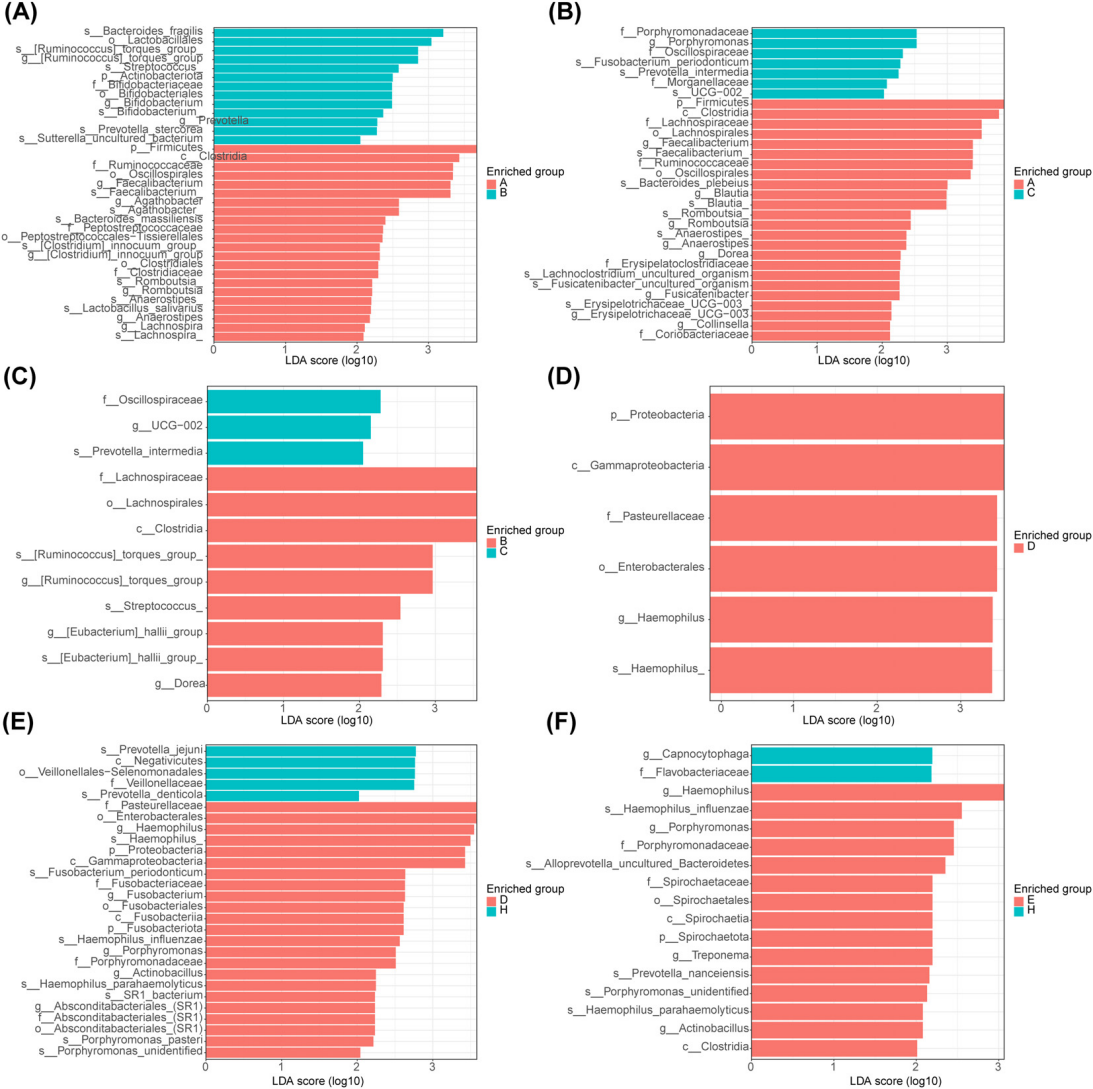
Linear discriminant analysis of saliva and fecal microbiota. **(A)** Analysis of significant species differences in feces between normal (group A) and adenoma (group B). **(B)** Analysis of significant species differences in feces between normal (group A) and CRC (group C). **(C)** Analysis of significant species differences in feces between adenoma (group B) and CRC (group C). **(D)** Analysis of significant species differences in saliva **(A)** between normal (group D) and adenoma (group E). **(E)** Analysis of significant species differences in saliva between normal (group D) and CRC (group F). **(F)** Analysis of significant species differences in saliva between adenoma (group E) and CRC (group F).

After analysis between group A and group B, the main microbiota in group A consisted of 22 species (Firmicutes, Ruminococcaceae, etc.) and in group B there were 13 species (Bacteroides fragilis, Lactobacillales, etc.) (**Figure 4A** **,** **Supplementary Table 1**). Whereas between groups A and C, the important flora in group A included 24 species such as Firmicutes, Ruminococcaceae, etc., and 7 species such as Porphyromonadaceae, Porphyromonas, etc. in group C (**Figure 4B** **,** **Supplementary Table 2**). However, between groups B and C, there were 9 important microbiota (Lachnospirales, Streptococcus, etc.) in group B and three (Oscillospiraceae, UCG−002 and Prevotella intermedia) in group C (**Figure 4C** **,** **Supplementary Table 3**). After analysis between group D and group E, the main microbiota in group D consisted of 6 species (Gammaproteobacteria, Proteobacteria, etc.), while no species were identified in group E (**Figure 4D** **,** **Supplementary Table 4**). Whereas between groups D and H, the important flora in group D included 23 species such as Enterobacterales, Pasteurellaceae, etc., and 5 species such as Negativicutes, Prevotella jejun, etc. in group H (**Figure 4E** **,** **Supplementary Table 5**). However, between groups E and H, there were 23 important microbiota (Hemophilus, Porphyromonas, etc.) in group E and 2 (Capnocytophaga and Flavobacteriaceae) in group H (**Figure 4F** **,** **Supplementary Table 6**).

**Figure 5:**
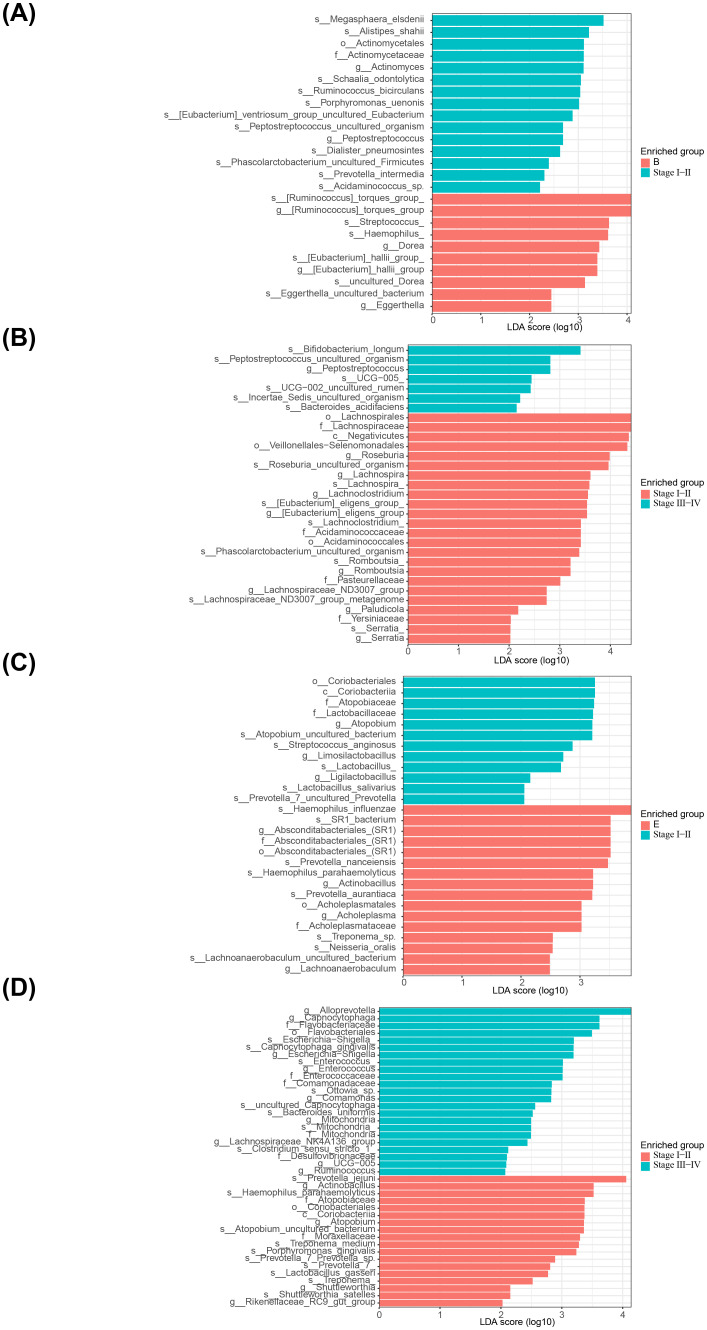
Linear discriminant analysis of saliva and fecal microbiota. **(A)** Analysis of significant species differences in feces between adenoma group and early CRC group. **(B)** Analysis of significant species differences in feces between early CRC group and late stage group. **(C)** Analysis of significant species differences in saliva between adenoma group and early CRC group. **(D)** Analysis of significant species differences in saliva between early CRC group and late stage group.

In addition, there were 15 important microbiota (Megasphaera elsdenii, Alistipes shahii, Actinomyces, etc.) in the feces for adenomas and 10 (Streptococcus, Hemophilus, Dorea, Eggerthella, etc.) for early stage CRC (**Figure 5A** **,** **Supplementary Table 7**). Whereas between early and late stage CRC, the important flora in early group included 7 species such as Bifidobacterium longum, Peptostreptococcus, etc., and 24 species such as Lachnospirales, Negativicutes, etc. in late stage group (**Figure 5B** **,** **Supplementary Table 8**). After analysis of adenoma and early CRC samples in saliva, the main microbiota in adenoma group consisted of 22 species (Coriobacteriales, Atopobiaceae, etc.) and in early CRC group there were 13 species (Hemophilus influenzae, SR1 bacterium, etc.) (**Figure 5C** **,** **Supplementary Table 9**). Finally, in saliva, there were 23 important flora (Alloprevotella, Capnocytophaga, etc.) in the early stage group of CRC and 18 (Actinobacillus, Atopobiaceae, etc.) in the late stage group (**Figure 5D** **,** **Supplementary Table 10**).

### 3.5 The diagnostic models of feature OTUs had good performance

**Figure 6:**
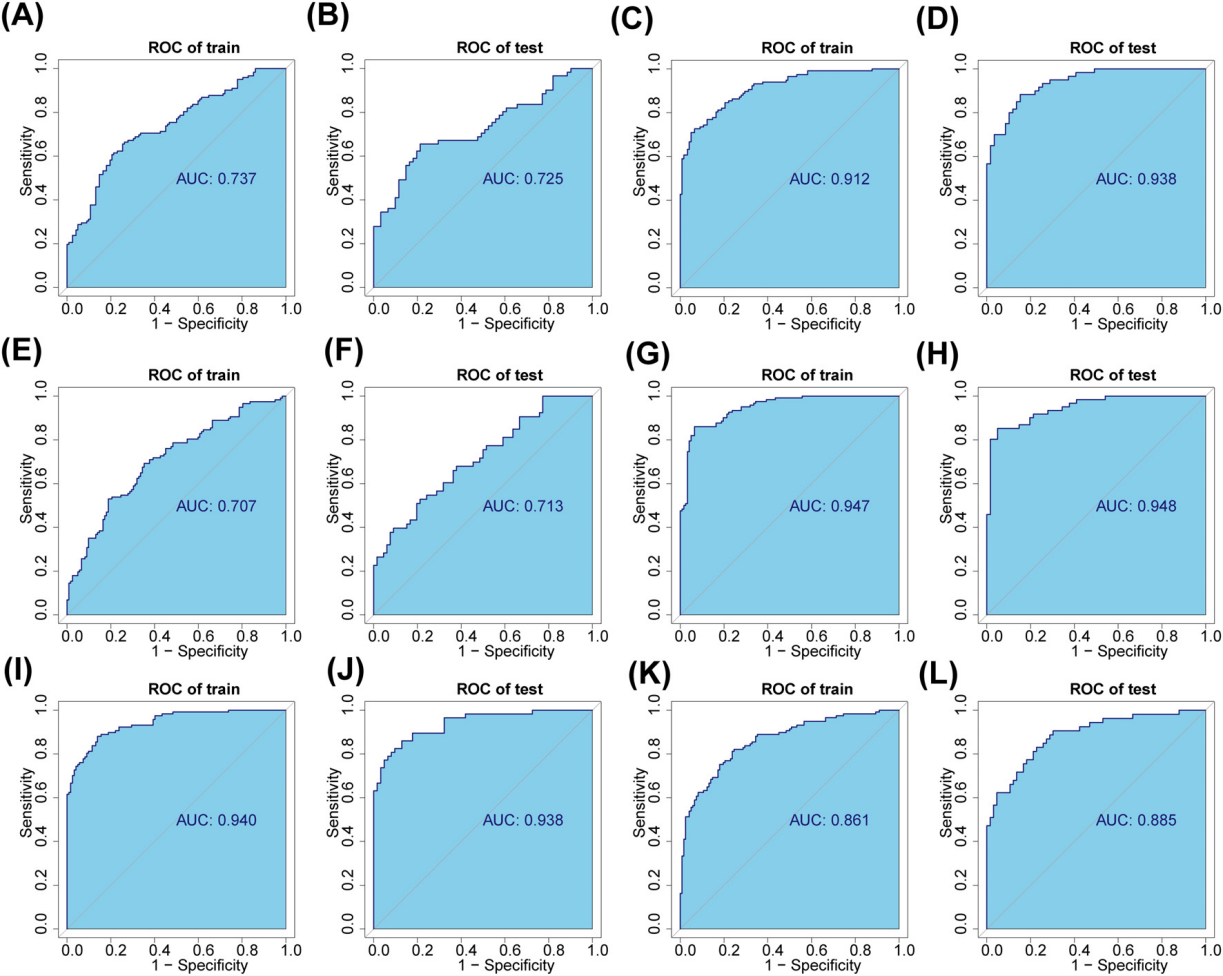
Saliva and fecal microbiota signatures distinguished the three groups. **(A, B)** The fecal characteristic OTUs of normal (group A) and adenoma (group B) were screened and verified. **(C, D)** The fecal characteristic OTUs of normal (group A) and CRC (group C) were screened and verified. **(E, F)** The fecal characteristic OTUs of adenoma (group B) and CRC (group C) were screened and verified. **(G, H)** The salivary characteristic OTUs of normal (group D) and adenoma (group E) were screened and verified. **(I, J)** The salivary characteristic OTUs of normal (group D) and CRC (group F) were screened and verified. **(K, L)** The salivary characteristic OTUs of adenoma (group E) and CRC (group F) were screened and verified.

In total, 9 AB-feature OTUs (ASV1, ASV13, ASV88, ASV113, ASV131, ASV268, ASV316, ASV644 and ASV651) were acquired in the A and B group by LASSO analysis (**Supplementary Figures 1A, B** **)**. The AUC values for both the training and validation sets were greater than 0.7 (**Figures 6A, B** **)**. A total of 43 AC-feature OTUs (ASV3, ASV7, ASV82, etc.) and 4 BC-feature OTU (ASV12, ASV104, ASV129 and ASV268) were gained (**Supplementary Figures 1C–F**). And the AUC values were all greater than 0.9 (**Figures 6C–F**). (**Supplementary Figures 1A–F**). In total, 52 DE-feature OTUs (ASV3, ASV194, etc.), 50 DH-feature OTUs (ASV323, ASV393, etc.) and 27 EH-feature OTUs (ASV22, ASV32, etc.) were obtained (**Supplementary Figures 1G–L**). And the AUC values were all greater than 0.85 (**Figures 6G–L**). All of the above studies had significant differences in POD analysis (**Supplementary Figures 2A–L**).

### 3.6 A total of 17 core-shared OTUs were identified

**Figure 7:**
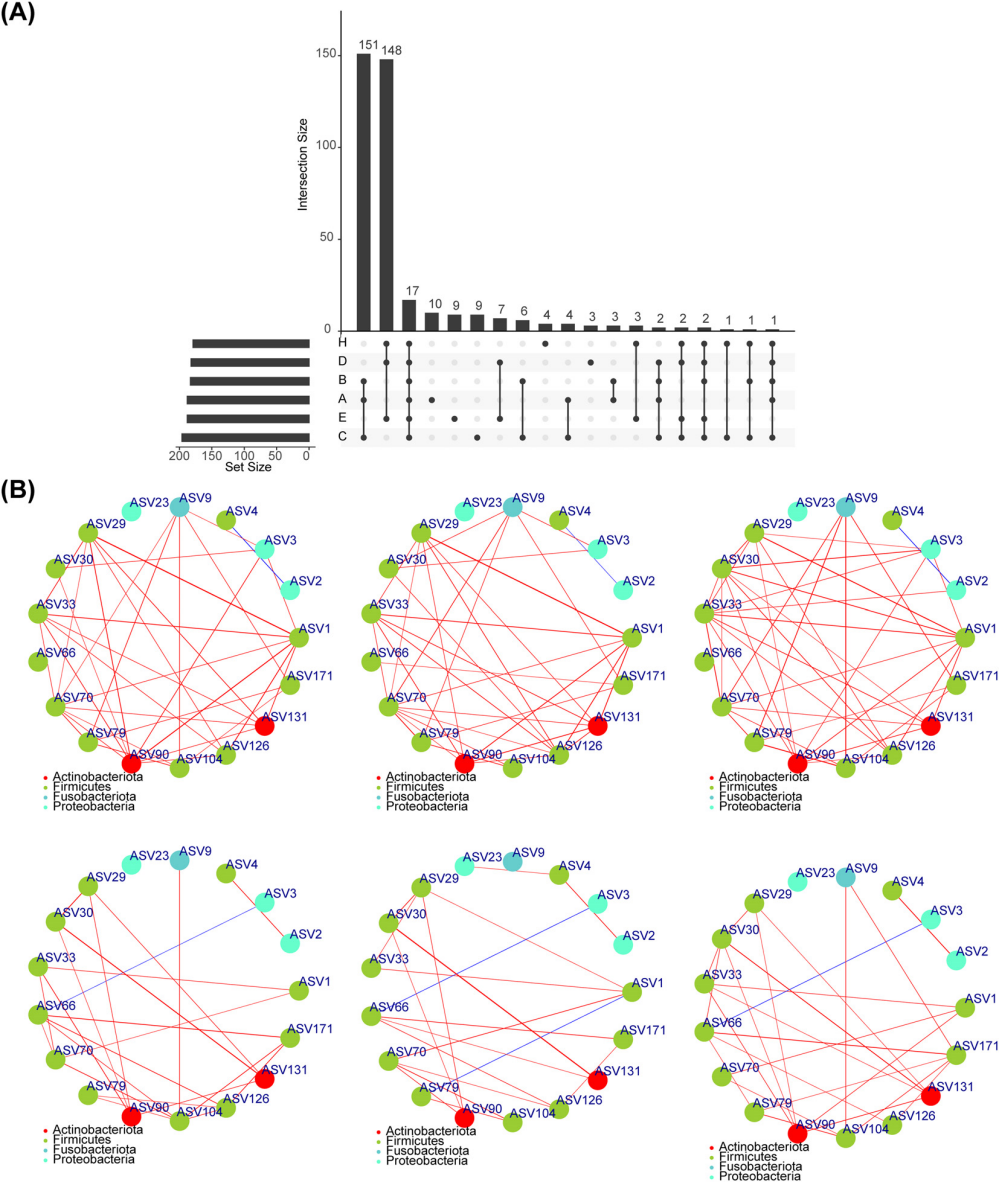
Core shared OTU. **(A)**Screening the OTU shared by normal, adenoma and CRC in feces and saliva, and obtaining a total of 17 shared OTUs. **(B)** Spearman correlation analysis between 17 shared OUTs showed that the microbial network in fecal group was more complicated than that in saliva group.

In total, 17 core-shared OTUs (ASV1, ASV2, ASV3, etc.) were acquired, and the results of the corresponding classification levels were shown in **Supplementary Table 11** (**Figure 7A**). The results of the core-shared OTU network for each group were illustrated in **Figure 7B** **,** with the network of microorganisms in the fecal group being more complex than that of the saliva group.

### 3.7 The correlation between different flora and clinical features

**Figure 8:**
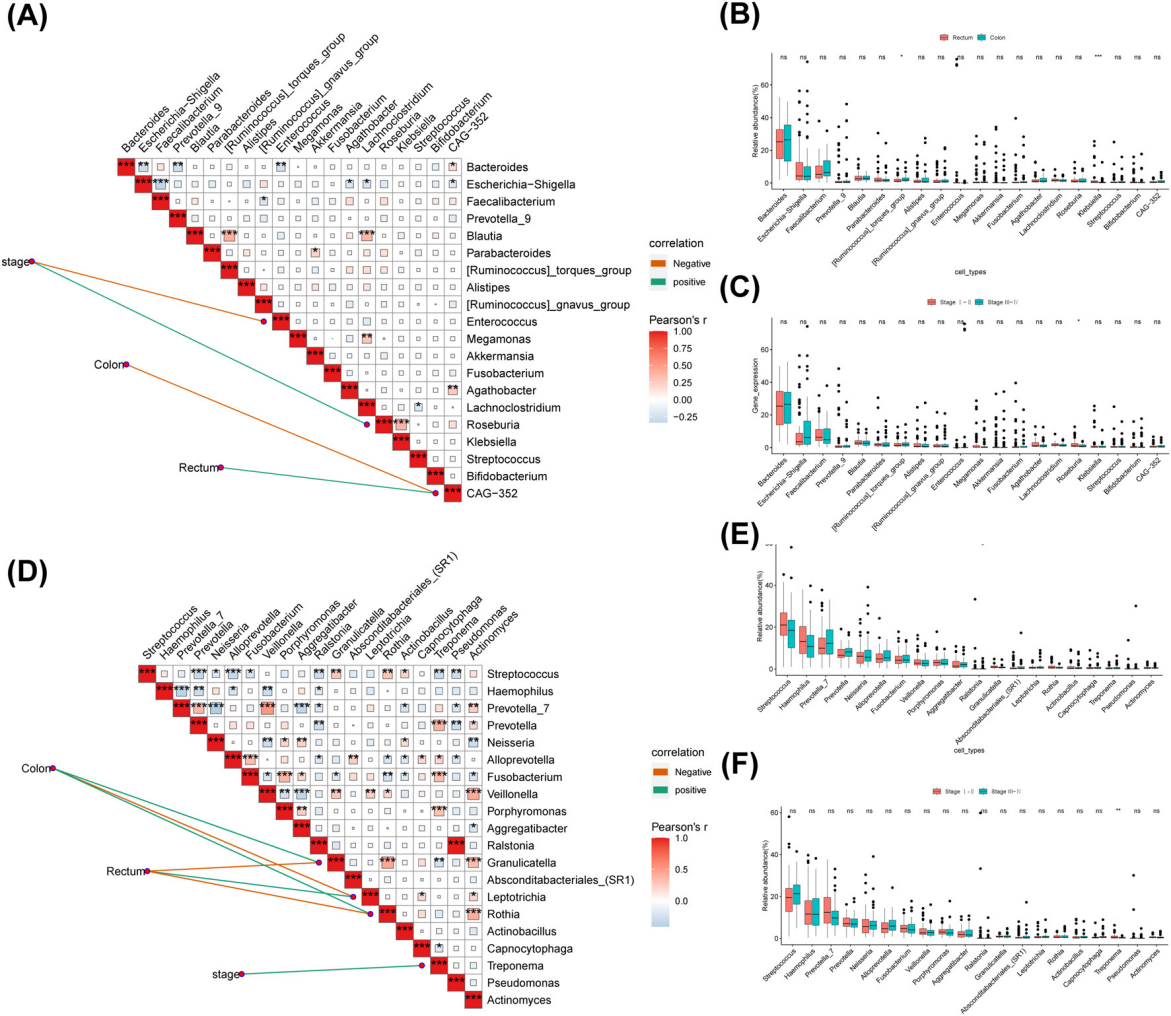
The correlation between different flora and clinical features. **(A)**The top 20 genera with the highest relative abundance were selected to calculate the correlation analysis between different clinical groups and the genus level of intestinal microbial community. **(B)** Analysis on the difference of fecal genus level in different cancer sites. **(C)** Analysis on the difference of fecal genus level in different Stage. **(D)** The top 20 genera with the highest relative abundance were selected to calculate the correlation analysis between different clinical groups and the genus level of saliva microbial community. **(E)** Analysis on the difference of saliva genus level in different cancer sites. **(F)** Analysis on the difference of saliva genus level in different Stage. ns, no significance; *: p < 0.05; **: p < 0.01; ***: p < 0.001.

The correlation analysis revealed that in the fecal group, stage was negatively correlated with Enterococcus and positive correlated with Roseburia, while Roseburia had a positively correlation with Klebsiella (**Figure 8A**). And CAG-352 had a negative association with the Colon and a positive association with the Rectum (**Figure 8B**). The results of the statistical analysis of differences indicated that [Ruminococcus] torques group and Klebsiella differed in cancer sites (**Figure 8B**). Roseburia differed significantly by stage and the proportion was greater in the early stages than in the late stages (**Figure 8C**). In the saliva, group stage exhibited a positive correlation with Treponema (**Figure 8D**). In addition, Granulicatella and Rothia were positively associated with Colon and negatively related to Rectum (**Figure 8D**). The colon was adversely associated with Leptotrichia and the rectum was positively related to Leptotrichia, while the opposite was true for Rothia (**Figure 8D**). The results of the difference analysis indicated a difference in cancer sites for Granulicatella and in stage for Treponema (**Figures 8E, F** **)**.

### 3.8 The microbial communities between different groups played a role in different signaling pathways

**Figure 9:**
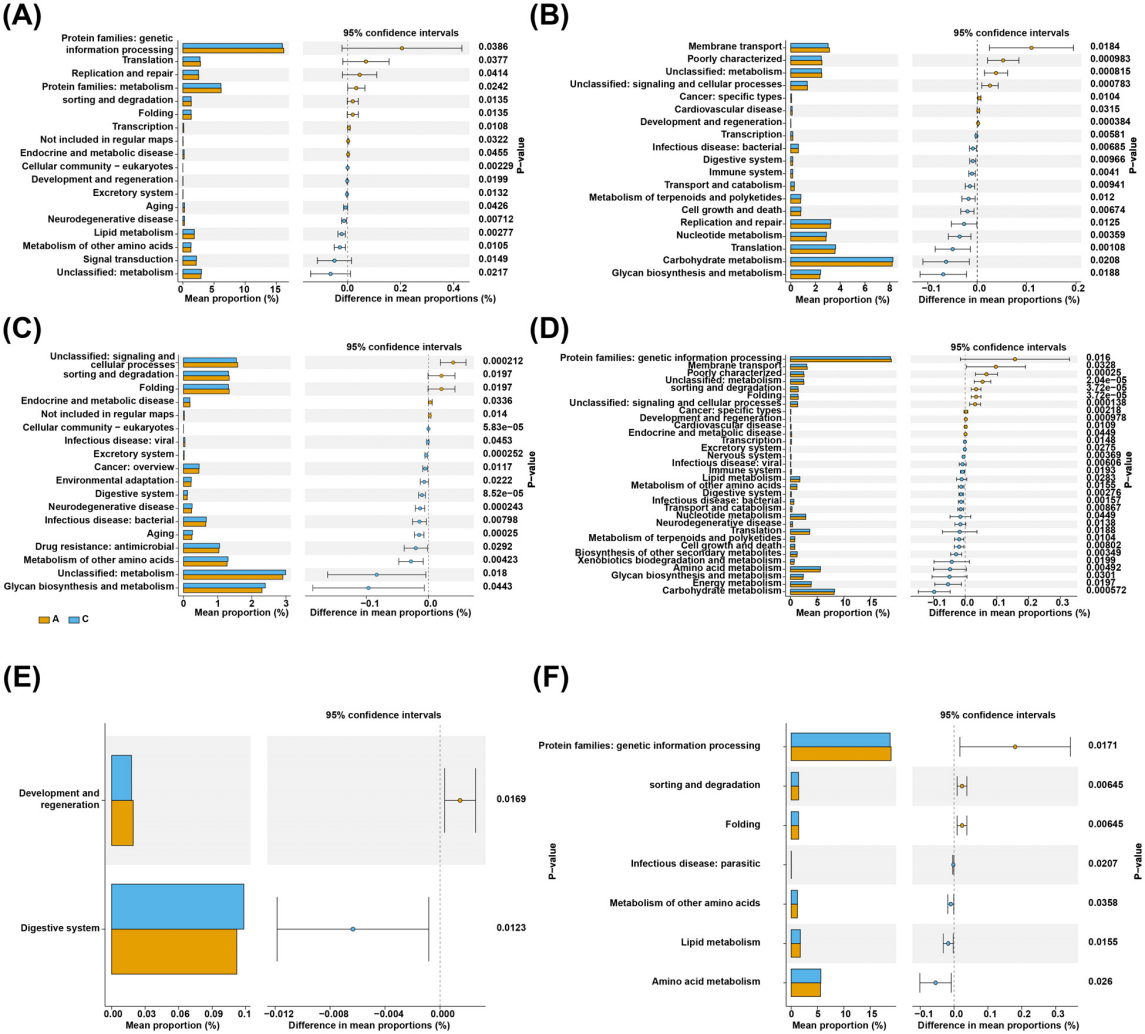
Functional enrichment analysis. On the left is the histogram of the relative abundance of different channel body entries in different groups, and the middle part is the abundance of channels in different branch groups. **(A)** A total of 18 KEGG pathways were significantly different between normal and adenoma fecal groups. **(B)** A total of 18 KEGG pathways were significantly different between normal and colorectal cancer fecal groups. **(C)** Only 2 KEGG pathways were significantly different between adenoma and intestinal cancer fecal group. **(D)** A total of 19 KEGG pathways were significantly different between normal and adenoma saliva groups. **(E)** A total of 19 KEGG pathways were significantly different between normal and colorectal cancer saliva groups. **(F)** Only 7 KEGG pathways were significantly different between adenoma and intestinal cancer saliva group.

The results of the enrichment analysis indicated that 18 different KEGG pathways were implicated in the differential flora of groups B and A, 18 in groups A and C, 2 in groups B and C, 19 in groups D and E, 32 in groups D and H, and 7 in groups E and H. Analysis of the functional differences between groups B and A revealed that Protein families: genetic information processing, Translation, etc. were highly enriched in group A, and Cellular community-eukaryotes, Development and regeneration, etc. were highly enriched in group B (**Figure 9A** **,** **Supplementary Table 12**). A and C groups enrichment results illustrated that the enrichment of Unclassified: signaling and cellular processes, sorting and degradation, Folding, Endocrine and metabolic disease and Not included in regular maps was significantly higher in group A than in group C (**Figure 9B** **,** **Supplementary Table 13**). Followed by, a total of 2 pathways (Development and regeneration and Digestive system) were significantly different between B and C groups, with Development and regeneration being at a higher level in group B (**Figure 9C** **,** **Supplementary Table 14**). Altogether 19 KEGG pathways were significantly different between groups D and E, Membrane transport, Poorly characterized, etc. were more plentiful in group D, and Transcription, Infectious disease: bacterial, etc. were more abundant in group E (**Figure 9D** **,** **Supplementary Table 15**). Analysis of the functional differences between groups D and H revealed that Protein families: genetic information processing, Membrane transport, etc. were highly enriched in group A, and Transcription, Excretory system, etc. were highly enriched in group H (**Figure 9E** **,** **Supplementary Table 16**). Finally, a total of 7 pathways were significantly different between E and H groups, with Protein families: genetic information processing, sorting and degradation and Folding being at a higher level in group E (**Figure 9F** **,** **Supplementary Table 17**).

## 4 Discussion

CRC is now the third most common cancer worldwide and the second leading cause of cancer death worldwide (18). Previous studies have demonstrated that an imbalance of the intestinal microbiota microecosystem can promote the development of CRC (19). The oral microorganisms are able to colonize the intestine and work with the intestinal flora to cause disease, so patients with CRC, etc. are rich in the intestines of a variety of microorganisms from the oral cavity (20, 21). However, there are few studies on the development of CRC by combined oral and intestinal microbiota analysis, and most of the relevant studies have been conducted in Western populations with very different dietary habits from those of China. In this study, the intestinal and oral microorganisms of adenoma and CRC in Yunnan region of China were analyzed for the first time, and the relationship between saliva flora and intestinal flora in normal-adenoma-CRC patients was initially explored, and a diagnostic model based on oral and fecal microbial markers was built to screen for biomarkers. The model can distinguish CRC, adenoma and healthy control groups, and realize strong classification potential in the training set and validation set, which provides a theoretical basis for the early screening and pathogenesis of CRC.

The Venn diagram showed that there were differential flora in fecal and saliva samples in the three groups, and there were more unique OTU numbers in the CRC group in saliva and feces compared with the normal and adenoma groups. In order to study the microbiota correlation of saliva and fecal samples in HC, adenoma and CRC, and a total of 17 core shared OTUs were screened in six groups. The exchange of genetic material between the core microbiota and the elastic microbiota gives the host the ability to adapt to the environment (22). Through Spearman correlation analysis, the microbial network plots of each group showed that the microbial network of the fecal group was more complex than that of the saliva group.

About 90% of the normal human intestinal flora is composed of Firmicutes, Bacteroidetes, and Proteobacteria, and less than 10% of Actinobacteria and Fusobacteria (23). More than 700 species of bacteria colonize the oral cavity, and more than 94% of the oral flora is also composed of these five phyla (24), and our study also found that feces and saliva at the phylum level are composed of these five phyla. Bacteroidetes are abundant in the feces of CRC patients compared with healthy individuals and is positively correlated with the development of CRC, and previous studies have come to the same conclusion (25). At the genus level, although the composition of oral and intestinal flora is very different, there are common bacteria (Streptococcus, Prevotella, Fusobacterium and other bacteria in the mouth and intestine). Nakatsu et al. (19) studied the characteristics of the gut microbial community of healthy humans with adenomas and CRCs, and detected a large number of bacteria derived from the oral cavity, including Fusobacterium and Streptococcus. It suggests that dynamic symbiotic communities are closely related to the occurrence of CRC. This study found that the microbial community showed early signs of dysregulation in adenomas during the development of normal-adenoma-CRC, and Fusobacterium and Escherichia-Shigella showed an increasing trend in feces, and found that Streptococcus also followed this rule in saliva. Microorganisms such as Escherichia coli and Fusobacterium destroy the intestinal barrier lining and colonic cell DNA, increase pro-inflammatory cytokines and oxidation factors, and produce potential carcinogenic toxins, which may become an important target for CRC therapy (11). Similarly, streptococcus in saliva is enriched in CRC (26), proving that with the occurrence and development of CRC, the number of pathogenic bacteria in the oral cavity and intestine increases.

At present, dozens of bacteria related to the pathogenesis of CRC have been confirmed (27). There is increasing evidence that pathogenic bacteria such as Fusobacterium nucleatum and Enterotoxigenic Bacteroides fragilis are associated with the occurrence of CRC (28, 29). In our study, we found that Fusobacterium gradually increased in abundance in the development and development of normal-adenoma-CRC (P < 0.05), which is consistent with previous studies that believed that Fusobacterium was enriched in the intestines of CRC patients (30), and Fusobacterium was also found to be enriched in the oral cavity of CRC patients, and it was believed that the Fusobacterium enriched in the intestines of CRC patients may be derived from the oral cavity (28, 31, 32). Analysis of salivary flora in our study found Fusobacterium_periodonticum that were enriched in the feces of CRC patients, but not detected in the normal and adenoma groups, presumably metastasizing from the mouth to the intestine and involved in the development of CRC, suggesting that Fusobacterium_periodonticum may be a “driver” for the development of colorectal CRC.

In the analysis of oral and intestinal microbiota diversity, there were significant differences in observed OTU index and PD index in the three groups. This indicates that the diversity and richness of the gut microbiota of CRC patients is increased compared with healthy controls (33). However, the current results are inconsistent, and some studies have found that gut microbial diversity is reduced in CRC (34). We speculate that the increase in diversity may be due to a significant increase in pathogenic bacteria. In saliva, there was a significant difference in species richness (P < 0.05) between adenomas and CRCs alone, with the most diversity in the adenoma group, consistent with published studies (31). In addition, significant differences in fecal and saliva microbiota β diversity between groups demonstrated that the differences between the three groups were significantly larger than within groups, and that the grouping was justified, with the most significant differences in saliva and feces in the normal and CRC groups, consistent with previous studies (26).The oral cavity is an open environment, and the intestinal environment may be more stable, selectively allowing certain microorganisms to survive. As the starting organ of the digestive tract, the oral cavity can colonize the intestine and interact with the intestinal flora to cause diseases. Intestinal colonization can be mediated by translocation of the oral microbiota, an oral-colonic link that allows the salivary microbiota to influence the development of the gut microbiota in some ways.

Selecting a single gut microbiome as a diagnostic marker is challenging (35) We characterized specific oral and gut microbial markers to distinguish adenoma or CRC patients from healthy controls, and validated their diagnostic efficacy using diagnostic models. The results show that the classifier based on the three best OTU markers in feces can effectively distinguish adenomas from healthy controls in the training and validation sets, namely Streptococcus, Bacteroides_fragilis, and Ruminococcus_bicirculans. Similarly, 7 microbiota, including Hemophilus, Faecalibacterium, Blautia, Romboutsia, Collinsella, Dorea, and UCG-002, were screened for diagnostic potential in CRC. In the differential microbiota analysis, Faecalibacterium, Blautia, Roseburia and other butyrate-producing bacteria are found to be significantly reduced in CRC patients and can separate the normal population and CRC patients with great predictive value. The butyrate-producing bacteria can produce short-chain fatty acids that affect colon movement, have anti-inflammatory properties, are enrich in the human intestine and has been shown to reduce the risk of CRC (34, 36, 37). Streptococcus, Bacteroides_fragilis, Hemophilus, etc. are closely related to the occurrence and development of CRC, which is consistent with the conclusions of many previous studies (38). Dorea and Ruminococcus_bicirculans have been reported to be reduced in the feces of CRC patients (39) and may contribute to the development of CRC as potential probiotic and antimicrobial agents. The reduction of collinsella and UCG-002 is the first time we have reported this. Collinsella is a bacterium found to be highly abundant in the intestines of patients with nonalcoholic steatohepatitis and type 2 diabetes (40), but its association and significance with CRC need further study. By analyzing the differences at the phylum and genus levels beta diversity, we can preliminarily determine the differences in microbial communities between different groups. LEFSe was used to screen for species with significant differences in saliva and intestinal microbiota abundance between normal, adenoma and bowel cancer groups. During the development of normal-adenoma-colorectal cancer, the number of Fusobacterium spp. and Escherichia coli-Shigella showed an increasing trend in feces, and the differential microbiota analysis showed that the number of butyrate-producing bacteria such as Faecalibacterium, Myxomyxobacterium and Romburgsa was significantly reduced in colorectal cancer patients, which could separate the normal population from colorectal cancer patients.

In this study, we identified two oral bacteria (Prevotella_jejuni and Hemophilus) that may be potential biomarkers for diagnosing CRC in saliva. The study by Flemer et al. (41) also found significant differences in oral Hemophilus and Prevotella between CRC patients and healthy people, suggesting that oral microbiota-based biomarkers may help predict the risk of adenomas and CRC. In recent years, more and more people attention has been paid to the intestinal flora at different stages of CRC to distinguish between early and late flora (17). Yachida et al. (42) found from analysis of stool samples from CRC patients at different stages that the abundance of intestinal microbiota in CRC patients increased progressively from early to late stages of the disease, including Fusobacterium nucleatum, Solobacterium moorei, and Peptostreptococcus stomatis. The correlation analysis between microbiota level and CRC stage revealed a significant increase in pathogenic bacteria Enterococcus and Klebsiella in the fecal group of intermediate to late CRC compared with early CRC. The study of WangT et al. (43) has found that Enterococcus and Klebsiella are enriched in the intestinal feces of CRC patients. In the salivary flora, Treponema showed a significant decrease as CRC progresses, and Ayeni et al. (36) found that Treponema is a bacterium present in normal humans with a predominantly fibrous digestive function. We report for the first time a significant reduction of Treponema in saliva of patients with advanced CRC, the significance of which needs to be further studied. Identification of driver strains related with the development of pathological stages of CRC may have implications for early screening for prevention of large CRC.

We performed a functional analysis using PICRUSt2. The results showed that a total of 18 KEGG pathways in feces are significantly different between normal and adenomas, with 10 KEGG pathways being increased in normal subjects, which are closely related to metabolism and genetic information processing. The 13 KEGG pathways increased in the CRC group, such as excretory system, cancer, drug resistance, aging, and were closely related to inflammation and immunity, and it was speculated that the intestinal flora may promote the occurrence and development of tumors by affecting metabolic pathways. The microbial function pathways of the normal group were significantly down-regulated in energy metabolism, cell motility, replication and repair, and up-regulated in amino acid metabolism and glucose metabolism, and the intestinal microbiome of colorectal cancer patients showed changes in functional enrichment in metabolism, which may lead to the production of more intestinal toxins and carcinogenic metabolites. At the same time, these changes may affect the immune system’s ability to monitor and eliminate bowel cancer cells.

At present, colonoscopy screening is still an effective method for detection large CRC to reduce its morbidity and mortality (44). With the potential of saliva and intestinal flora as a new generation of biomarkers has been further explored (45, 46). The establishment of early screening and diagnostic models of CRC based on microbial markers will facilitate the early detection of CRC, and the development of drugs with microorganisms as targets, which may become a new strategy for CRC prevention and treatment in the future. This study analyzes the fecal and salivary flora of adenoma and bowel cancer patients in Yunnan region of China for the first time, characterizes specific oral and intestinal microbial markers to distinguish adenoma or bowel cancer patients from healthy controls, and verifies their diagnostic efficacy using diagnostic models. Oral Fusobacterium periodonticum was found to be enriched in the feces of patients with bowel cancer, but not detected in the normal and adenoma groups, and it was speculated that the bacteria metastasized from the mouth to the intestine and participated in the occurrence of bowel cancer. This study analyzed for the first time the intestinal and oral microbes of adenoma and intestinal cancer in Yunnan, China, which is a plateau region with a large number of ethnic minorities and a rich food culture with a wide selection of ingredients and many flavors, and explored the composition, richness, diversity and differences of saliva and intestinal flora. Some ethnic minorities were selected for this study, and the results may not be fully representative of the entire Yunnan region or the broader population. At the same time, different countries, regions, different ethnic groups, races and dietary habits will have a great impact on microorganisms, and the results of studies may not be representative of the general population. Therefore, it is necessary to further expand the scope of research objects in the future, and at the same time carry out more multi-center studies to explore the feasibility of promoting the relevant research results in different regions.

## 5 Conclusion

In conclusion, 9 AB-feature OTUs, 43 AC-feature OTUs, 4 BC-feature OTUs, 52 DE-feature OTUs, 50 DH-feature OTUs and 27 EH-feature OTUs were obtained by LASSO regression analysis. Then, 17 core-shared OTUs (ASV1, ASV2, ASV3, etc.) were acquired among the saliva and fecal samples of normal control, adenoma, and colorectal cancer. In addition, the dominant flora was remarkably different between groups and between periods. Thus, the results of this study could provide an important reference for differentiating between normal, adenoma and CRC patients.

## Data Availability

The original contributions presented in the study are included in the article/**Supplementary Material**. Further inquiries can be directed to the corresponding authors.
