# Supplementary material for: Dysregulation of saliva and fecal microbiota as novel biomarkers of colorectal cancer
Source: Front Oncol. 2024 Dec 18;14:1498328. doi: 10.3389/fonc.2024.1498328 (PMC11688226; doi:10.3389/fonc.2024.1498328)
Supplement: Supplementary file 1 [file DataSheet1.pdf]

Supplementary Material

1 Supplementary Figures and Tables

1.1 Supplementary Figures

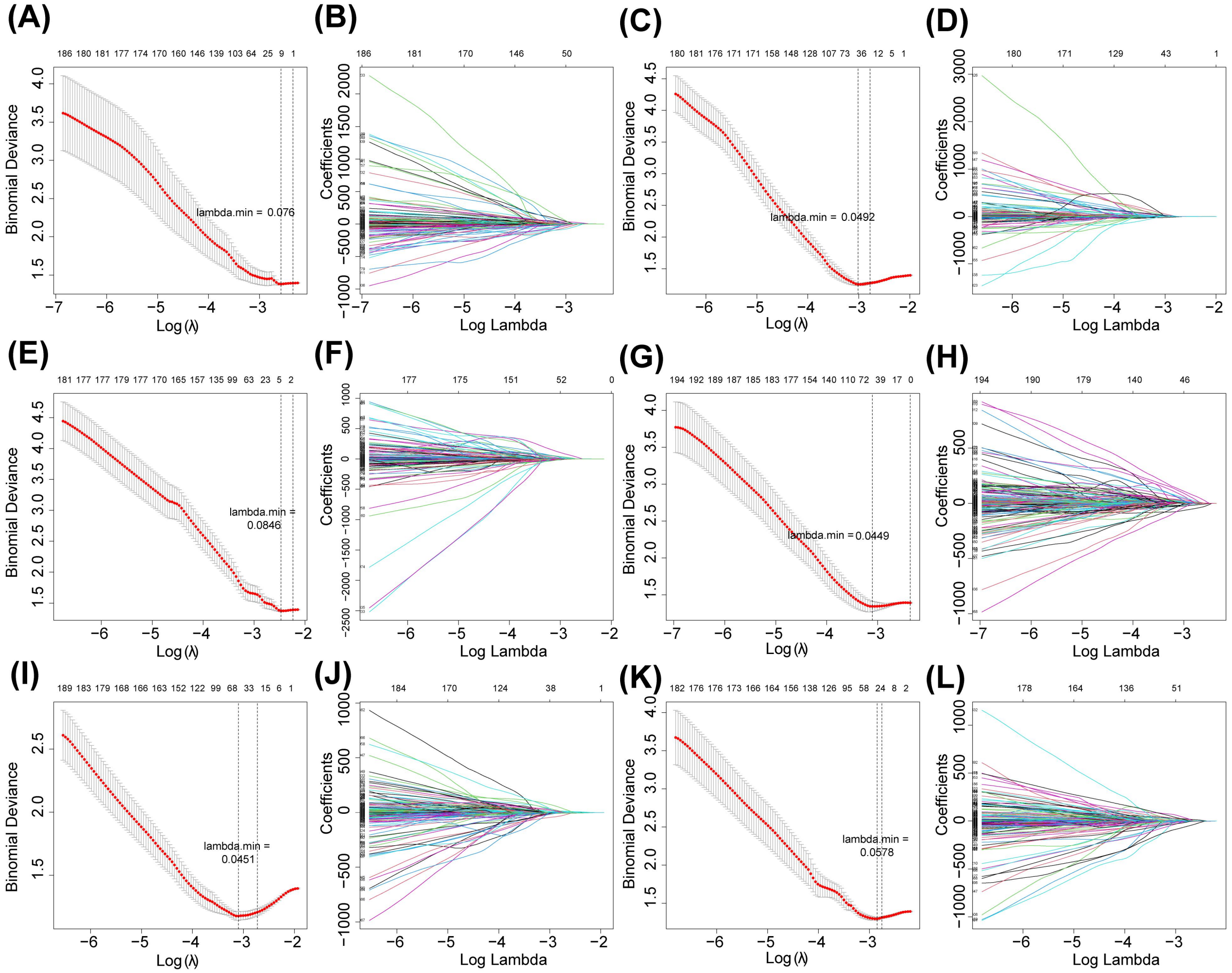

Supplementary Figure 1. Screening of variables based on Lasso regression. **(A)**, **(C)**, **(E)**, **(G)**, **(I)**, **(K)** The variation characteristics of the coefficient of variables. **(B)**, **(D)**, **(F)**, **(H)**, **(J)**, **(L)** The selection process of the optimum value of the parameter l in the Lasso regression model by cross-validation method.

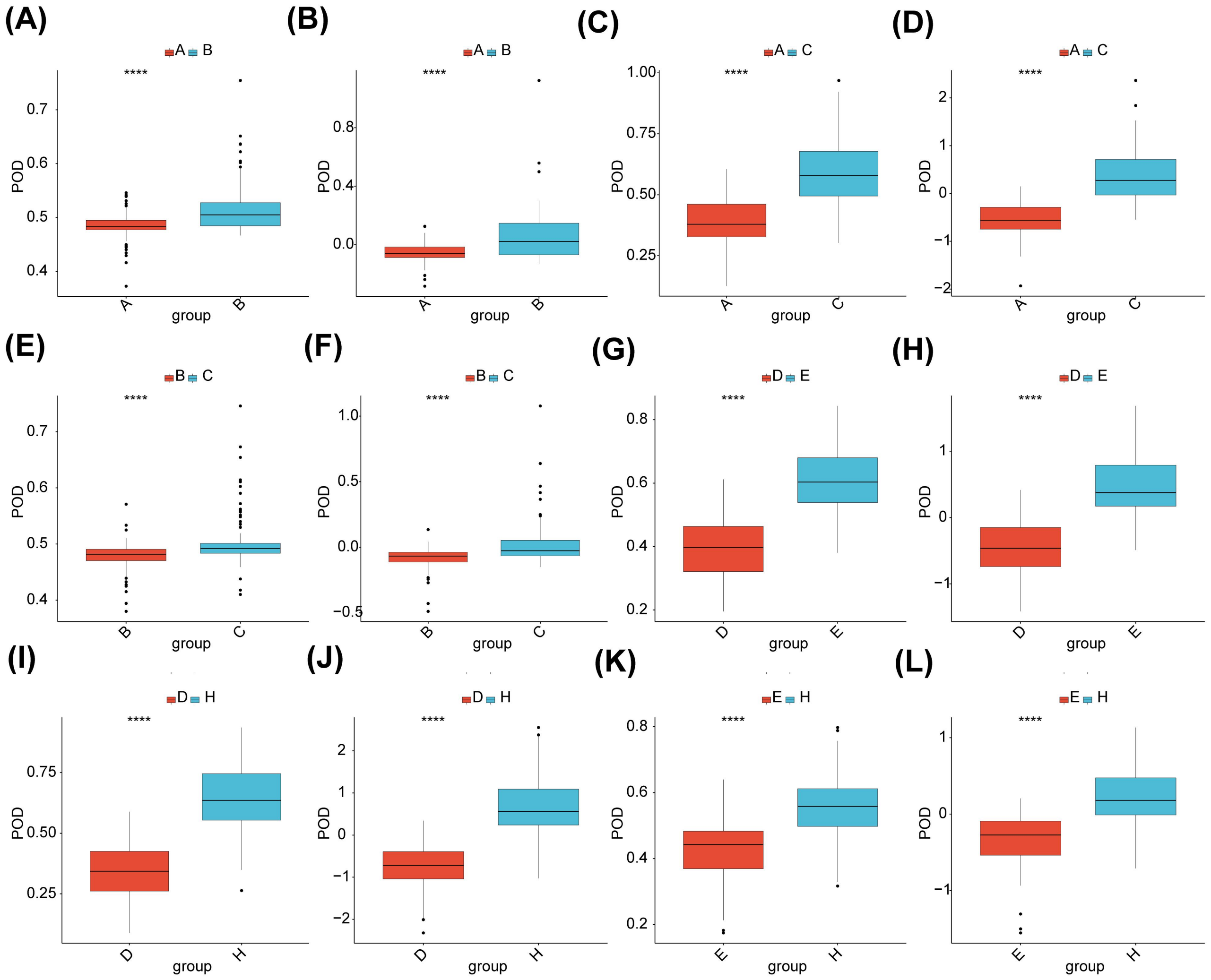

Supplementary Figure 2. POD analysis. **(A-L)** POD analysis between different groups.

1.2 Supplementary Tables

Supplementary Table 1. Analysis of difference in feces between normal (group A) and adenoma (group B).

	feature	enrich_group	ef_lda	pvalue	padj
marker 1	k__Bacteria|p__Firmicutes	A	3.674209878	0.026888454	0.026888454
marker 2	k__Bacteria|p__Firmicutes|c__Clostridia	A	3.428585315	0.043672913	0.043672913
marker 3	k__Bacteria|p__Firmicutes|c__Clostridia|o__Oscillospirales|f__Ruminococcaceae	A	3.341242746	0.000878002	0.000878002
marker 4	k__Bacteria|p__Firmicutes|c__Clostridia|o__Oscillospirales	A	3.339376758	0.001290367	0.001290367
marker 5	k__Bacteria|p__Firmicutes|c__Clostridia|o__Oscillospirales|f__Ruminococcaceae|g__Faecalibacterium	A	3.305790358	0.000822537	0.000822537
marker 6	k__Bacteria|p__Firmicutes|c__Clostridia|o__Oscillospirales|f__Ruminococcaceae|g__Faecalibacterium|s__Faecalibacterium_	A	3.305771941	0.000811842	0.000811842
marker 7	k__Bacteria|p__Firmicutes|c__Clostridia|o__Lachnospirales|f__Lachnospiraceae|g__Agathobacter	A	2.585001445	0.00284412	0.00284412
marker 8	k__Bacteria|p__Firmicutes|c__Clostridia|o__Lachnospirales|f__Lachnospiraceae|g__Agathobacter|s__Agathobacter_	A	2.584811361	0.00284412	0.00284412

marker 9	k__Bacteria|p__Bacteroidota|c__Bacteroidia|o__Bacteroidales|f__Bacteroidaceae|g__Bacteroides|s__Bacteroides_massiliensis	A	2.39553124	0.024711172	0.024711172
marker 10	k__Bacteria|p__Firmicutes|c__Clostridia|o__Peptostreptococcales-Tissierellales|f__Peptostreptococcaceae	A	2.364058892	0.002616793	0.002616793
marker 11	k__Bacteria|p__Firmicutes|c__Clostridia|o__Peptostreptococcales-Tissierellales	A	2.355017853	0.005011002	0.005011002
marker 12	k__Bacteria|p__Firmicutes|c__Bacilli|o__Erysipelotrichales|f__Erysipelotrichaceae|g__[Clostridium]_innocuum_group	A	2.318061323	0.015865378	0.015865378
marker 13	k__Bacteria|p__Firmicutes|c__Bacilli|o__Erysipelotrichales|f__Erysipelotrichaceae|g__[Clostridium]_innocuum_group|s__[Clostridium]_innocuum_group_	A	2.318061323	0.015865378	0.015865378
marker 14	k__Bacteria|p__Firmicutes|c__Clostridia|o__Clostridiales	A	2.29622909	0.049040724	0.049040724
marker 15	k__Bacteria|p__Firmicutes|c__Clostridia|o__Clostridiales|f__Clostridiaceae	A	2.296007066	0.049881209	0.049881209
marker 16	k__Bacteria|p__Firmicutes|c__Clostridia|o__Peptostreptococcales-Tissierellales|f__Peptostreptococcaceae|g__Romboutsia	A	2.211048572	0.000740515	0.000740515
marker 17	k__Bacteria|p__Firmicutes|c__Clostridia|o__Peptostreptococcales-Tissierellales|f__Peptostreptococcaceae|g__Romboutsia|s__Romboutsia_	A	2.211048572	0.000740515	0.000740515
marker 18	k__Bacteria|p__Firmicutes|c__Clostridia|o__Lachnospirales|f__Lachnospiraceae|g__Anaerostipes|s__Anaerostipes_	A	2.199150699	0.012946397	0.012946397
marker 19	k__Bacteria|p__Firmicutes|c__Bacilli|o__Lactobacillales|f__Lactobacillaceae|g__Ligilactobacillus|s__Lactobacillus_salivarius	A	2.193312339	0.017690219	0.017690219

marker 20	k__Bacteria|p__Firmicutes|c__Clostridia|o__Lachnospirales|f__Lachnospiraceae|g__Anaerostipes	A	2.178728665	0.030452486	0.030452486
marker 21	k__Bacteria|p__Firmicutes|c__Clostridia|o__Lachnospirales|f__Lachnospiraceae|g__Lachnospira	A	2.106759282	0.003107577	0.003107577
marker 22	k__Bacteria|p__Firmicutes|c__Clostridia|o__Lachnospirales|f__Lachnospiraceae|g__Lachnospira|s__Lachnospira_	A	2.089956754	0.004299554	0.004299554
marker 23	k__Bacteria|p__Bacteroidota|c__Bacteroidia|o__Bacteroidales|f__Bacteroidaceae|g__Bacteroides|s__Bacteroides_fragilis	B	3.206215466	0.002946954	0.002946954
marker 24	k__Bacteria|p__Firmicutes|c__Bacilli|o__Lactobacillales	B	3.039665725	0.030872515	0.030872515
marker 25	k__Bacteria|p__Firmicutes|c__Clostridia|o__Lachnospirales|f__Lachnospiraceae|g__[Ruminococcus]_torques_group|s__[Ruminococcus]_torques_group_	B	2.853484185	0.010755449	0.010755449
marker 26	k__Bacteria|p__Firmicutes|c__Clostridia|o__Lachnospirales|f__Lachnospiraceae|g__[Ruminococcus]_torques_group	B	2.85319079	0.010811553	0.010811553
marker 27	k__Bacteria|p__Firmicutes|c__Bacilli|o__Lactobacillales|f__Streptococcaceae|g__Streptococcus|s__Streptococcus_	B	2.581945952	0.026085219	0.026085219
marker 28	k__Bacteria|p__Actinobacteriota	B	2.497653333	0.033347591	0.033347591
marker 29	k__Bacteria|p__Actinobacteriota|c__Actinobacteria|o__Bifidobacteriales	B	2.489590033	0.007681958	0.007681958
marker 30	k__Bacteria|p__Actinobacteriota|c__Actinobacteria|o__Bifidobacteriales|f__Bifidobacteriaceae	B	2.489590033	0.007681958	0.007681958

marker31	k__Bacteria|p__Actinobacteriota|c__Actinobacteria|o__Bifidobacteriales|f__Bifidobacteriaceae|g__Bifidobacterium	B	2.489558507	0.007043882	0.007043882
marker32	k__Bacteria|p__Actinobacteriota|c__Actinobacteria|o__Bifidobacteriales|f__Bifidobacteriaceae|g__Bifidobacterium|s__Bifidobacterium_	B	2.365246735	0.00791227	0.00791227
marker33	k__Bacteria|p__Bacteroidota|c__Bacteroidia|o__Bacteroidales|f__Prevotellaceae|g__Prevotella	B	2.283688254	0.027969741	0.027969741
marker34	k__Bacteria|p__Bacteroidota|c__Bacteroidia|o__Bacteroidales|f__Prevotellaceae|g__Prevotella|s__Prevotella_stercorea	B	2.277109451	0.044770628	0.044770628
marker35	k__Bacteria|p__Proteobacteria|c__Gammaproteobacteria|o__Burkholderiales|f__Sutterellaceae|g__Sutterella|s__Sutterella_uncultured_bacterium	B	2.045452549	0.007142545	0.007142545

Supplementary Table 2. Analysis of difference in feces between normal (group A) and CRC (group C).

	feature	enrich_group	ef_lda	pvalue	padj
marker1	k__Bacteria|p__Firmicutes	A	3.875838439	8.97E-07	8.97E-07
marker2	k__Bacteria|p__Firmicutes|c__Clostridia	A	3.795114502	2.33E-07	2.33E-07
marker3	k__Bacteria|p__Firmicutes|c__Clostridia|o__Lachnospirales|f__Lachnospiraceae	A	3.529923708	0.000225211	0.000225211
marke	k__Bacteria|p__Firmicutes|c__Clostridia|o__Lachnospirales	A	3.529880	0.000225	0.000225

r4			844	211	211
marker5	k__Bacteria|p__Firmicutes|c__Clostridia|o__Oscillospirales|f__Ruminococcaceae|g__Faecalibacterium	A	3.393557602	3.17E-05	3.17E-05
marker6	k__Bacteria|p__Firmicutes|c__Clostridia|o__Oscillospirales|f__Ruminococcaceae|g__Faecalibacterium|s__Faecalibacterium_	A	3.393434708	3.20E-05	3.20E-05
marker7	k__Bacteria|p__Firmicutes|c__Clostridia|o__Oscillospirales|f__Ruminococcaceae	A	3.391565811	0.000171164	0.000171164
marker8	k__Bacteria|p__Firmicutes|c__Clostridia|o__Oscillospirales	A	3.360054591	0.000786099	0.000786099
marker9	k__Bacteria|p__Bacteroidota|c__Bacteroidia|o__Bacteroidales|f__Bacteroidaceae|g__Bacteroides|s__Bacteroides_plebeius	A	3.003174476	0.001345733	0.001345733
marker10	k__Bacteria|p__Firmicutes|c__Clostridia|o__Lachnospirales|f__Lachnospiraceae|g__Blautia	A	2.988383773	1.50E-05	1.50E-05
marker11	k__Bacteria|p__Firmicutes|c__Clostridia|o__Lachnospirales|f__Lachnospiraceae|g__Blautia|s__Blautia_	A	2.986650512	1.55E-05	1.55E-05
marker12	k__Bacteria|p__Firmicutes|c__Clostridia|o__Peptostreptococcales-Tissierellales|f__Peptostreptococcaceae|g__Romboutsia	A	2.437730356	3.20E-05	3.20E-05
marker13	k__Bacteria|p__Firmicutes|c__Clostridia|o__Peptostreptococcales-Tissierellales|f__Peptostreptococcaceae|g__Romboutsia|s__Romboutsia_	A	2.437730356	3.20E-05	3.20E-05
marker14	k__Bacteria|p__Firmicutes|c__Clostridia|o__Lachnospirales|f__Lachnospiraceae|g__Anaerostipes|s__Anaerostipes_	A	2.371698225	6.30E-06	6.30E-06

marker15	k__Bacteria|p__Firmicutes|c__Clostridia|o__Lachnospirales|f__Lachnospiraceae|g__Anaerostipes	A	2.370626768	4.82E-06	4.82E-06
marker16	k__Bacteria|p__Firmicutes|c__Clostridia|o__Lachnospirales|f__Lachnospiraceae|g__Dorea	A	2.282873459	0.000495753	0.000495753
marker17	k__Bacteria|p__Firmicutes|c__Bacilli|o__Erysipelotrichales|f__Erysipelatoclostridiaceae	A	2.276383848	0.000355613	0.000355613
marker18	k__Bacteria|p__Firmicutes|c__Clostridia|o__Lachnospirales|f__Lachnospiraceae|g__Lachnoclostridium|s__Lachnoclostridium_uncultured_organism	A	2.268699899	0.001915007	0.001915007
marker19	k__Bacteria|p__Firmicutes|c__Clostridia|o__Lachnospirales|f__Lachnospiraceae|g__Fusicatenibacter	A	2.267004238	0.000585763	0.000585763
marker20	k__Bacteria|p__Firmicutes|c__Clostridia|o__Lachnospirales|f__Lachnospiraceae|g__Fusicatenibacter|s__Fusicatenibacter_uncultured_organism	A	2.267004238	0.000585763	0.000585763
marker21	k__Bacteria|p__Firmicutes|c__Bacilli|o__Erysipelotrichales|f__Erysipelatoclostridiaceae|g__Erysipelotrichaceae_UCG-003	A	2.141334561	0.001473554	0.001473554
marker22	k__Bacteria|p__Firmicutes|c__Bacilli|o__Erysipelotrichales|f__Erysipelatoclostridiaceae|g__Erysipelotrichaceae_UCG-003|s__Erysipelotrichaceae_UCG-003_	A	2.141334561	0.001473554	0.001473554
marker23	k__Bacteria|p__Actinobacteriota|c__Coriobacteriia|o__Coriobacteriales|f__Coriobacteriaceae	A	2.121362437	0.001541577	0.001541577
marker24	k__Bacteria|p__Actinobacteriota|c__Coriobacteriia|o__Coriobacteriales|f__Coriobacteriaceae|g__Collinsella	A	2.121362437	0.001541577	0.001541577
marker25	k__Bacteria|p__Bacteroidota|c__Bacteroidia|o__Bacteroidales|f__Porphyromonadaceae	C	2.529608906	6.25E-05	6.25E-05

marker26	k__Bacteria|p__Bacteroidota|c__Bacteroidia|o__Bacteroidales|f__Porphyromonadaceae|g__Porphyromonas	C	2.529528348	6.25E-05	6.25E-05
marker27	k__Bacteria|p__Firmicutes|c__Clostridia|o__Oscillospirales|f__Oscillospiraceae	C	2.316476808	0.001277365	0.001277365
marker28	k__Bacteria|p__Fusobacteriota|c__Fusobacteriia|o__Fusobacteriales|f__Fusobacteriaceae|g__Fusobacterium|s__Fusobacterium_periodonticum	C	2.279849546	1.42E-06	1.42E-06
marker29	k__Bacteria|p__Bacteroidota|c__Bacteroidia|o__Bacteroidales|f__Prevotellaceae|g__Prevotella|s__Prevotella_intermedia	C	2.252373226	0.000390712	0.000390712
marker30	k__Bacteria|p__Proteobacteria|c__Gammaproteobacteria|o__Enterobacterales|f__Morganellaceae	C	2.071831688	0.000219208	0.000219208
marker31	k__Bacteria|p__Firmicutes|c__Clostridia|o__Oscillospirales|f__Oscillospiraceae|g__UCG-002|s__UCG-002_	C	2.02662475	0.000775501	0.000775501

Supplementary Table 3. Analysis of difference in feces between adenoma (group B) and CRC (group C).

	feature	enrich_group	ef_lda	pvalue	padj
marker1	k__Bacteria|p__Firmicutes|c__Clostridia|o__Lachnospirales|f__Lachnospiraceae	B	3.544794145	3.47E-05	3.47E-05
marker2	k__Bacteria|p__Firmicutes|c__Clostridia|o__Lachnospirales	B	3.544761679	3.50E-05	3.50E-05

marker 3	k__Bacteria|p__Firmicutes|c__Clostridia	B	3.540148951	0.00053901	0.00053901
marker 4	k__Bacteria|p__Firmicutes|c__Clostridia|o__Lachnospirales|f__Lachnospiraceae|g__[Ruminococcus]_torques_group|s__[Ruminococcus]_torques_group_	B	2.9635781	4.36E-05	4.36E-05
marker 5	k__Bacteria|p__Firmicutes|c__Clostridia|o__Lachnospirales|f__Lachnospiraceae|g__[Ruminococcus]_torques_group	B	2.96344202	4.43E-05	4.43E-05
marker 6	k__Bacteria|p__Firmicutes|c__Bacilli|o__Lactobacillales|f__Streptococcaceae|g__Streptococcus|s__Streptococcus_	B	2.540317745	0.000115411	0.000115411
marker 7	k__Bacteria|p__Firmicutes|c__Clostridia|o__Lachnospirales|f__Lachnospiraceae|g__[Eubacterium]_hallii_group	B	2.308444487	0.000104534	0.000104534
marker 8	k__Bacteria|p__Firmicutes|c__Clostridia|o__Lachnospirales|f__Lachnospiraceae|g__[Eubacterium]_hallii_group|s__[Eubacterium]_hallii_group_	B	2.308444487	0.000104534	0.000104534
marker 9	k__Bacteria|p__Firmicutes|c__Clostridia|o__Lachnospirales|f__Lachnospiraceae|g__Dorea	B	2.290021311	0.000103733	0.000103733
marker 10	k__Bacteria|p__Firmicutes|c__Clostridia|o__Oscillospirales|f__Oscillospiraceae	C	2.278226668	0.000436899	0.000436899
marker 11	k__Bacteria|p__Firmicutes|c__Clostridia|o__Oscillospirales|f__Oscillospiraceae|g__UCG-002	C	2.149195971	0.000665288	0.000665288
marker 12	k__Bacteria|p__Bacteroidota|c__Bacteroidia|o__Bacteroidales|f__Prevotellaceae|g__Prevotella|s__Prevotella_intermedia	C	2.048416765	0.000838078	0.000838078

Supplementary Table 4. Analysis of difference in saliva (A) between normal (group D) and adenoma (group E).

	feature	enric h_gro up	ef_ld a	pvalu e	padj
mar ker 1	k__Bacteria|p__Proteobacteria	D	3.518 7610 72	0.012 0513 72	0.012 0513 72
mar ker 2	k__Bacteria|p__Proteobacteria|c__Gammaproteobacteria	D	3.517 5388 52	0.013 2127 17	0.013 2127 17
mar ker 3	k__Bacteria|p__Proteobacteria|c__Gammaproteobacteria|o__Enterobacterales|f__Pasteurellaceae	D	3.434 2039 02	0.013 1456 92	0.013 1456 92
mar ker 4	k__Bacteria|p__Proteobacteria|c__Gammaproteobacteria|o__Enterobacterales	D	3.433 4278 48	0.014 0409 05	0.014 0409 05
mar ker 5	k__Bacteria|p__Proteobacteria|c__Gammaproteobacteria|o__Enterobacterales|f__Pasteurellaceae|g__Haemophilus	D	3.380 4679 31	0.016 5591 9	0.016 5591 9
mar ker 6	k__Bacteria|p__Proteobacteria|c__Gammaproteobacteria|o__Enterobacterales|f__Pasteurellaceae|g__Haemophilus|s__Haemophilus_	D	3.374 6953 42	0.009 6355 66	0.009 6355 66

Supplementary Table 5. Analysis of difference in saliva between normal (group D) and CRC (group F).

	feature	enric h_gr oup	ef_l da	pval ue	padj
ma rke	k__Bacteria|p__Proteobacteria|c__Gammaproteobacteria|o__Enterobacterales|f__Pasteurellaceae	D	3.58 1041	8.82 E-05	8.82 E-05

r1			862		
ma rke r2	k__Bacteria|p__Proteobacteria|c__Gammaproteobacteria|o__Ent erobacterales	D	3.57 5682 23	0.00 0134 507	0.00 0134 507
ma rke r3	k__Bacteria|p__Proteobacteria|c__Gammaproteobacteria|o__Ent erobacterales|f__Pasteurellaceae|g__Haemophilus	D	3.54 4739 67	2.76 E-05	2.76 E-05
ma rke r4	k__Bacteria|p__Proteobacteria|c__Gammaproteobacteria|o__Ent erobacterales|f__Pasteurellaceae|g__Haemophilus|s__Haemophil us_	D	3.49 6748 598	3.99 E-05	3.99 E-05
ma rke r5	k__Bacteria|p__Proteobacteria	D	3.42 5506 28	0.00 1602 171	0.00 1602 171
ma rke r6	k__Bacteria|p__Proteobacteria|c__Gammaproteobacteria	D	3.42 5320 51	0.00 1718 988	0.00 1718 988
ma rke r7	k__Bacteria|p__Fusobacteriota|c__Fusobacteriia|o__Fusobacteri ales|f__Fusobacteriaceae|g__Fusobacterium|s__Fusobacterium_p eriodonticum	D	2.63 5260 12	0.00 9641 803	0.00 9641 803
ma rke r8	k__Bacteria|p__Fusobacteriota|c__Fusobacteriia|o__Fusobacteri ales|f__Fusobacteriaceae	D	2.63 2128 85	0.00 8738 817	0.00 8738 817
ma rke r9	k__Bacteria|p__Fusobacteriota|c__Fusobacteriia|o__Fusobacteri ales|f__Fusobacteriaceae|g__Fusobacterium	D	2.63 2110 423	0.00 8643 303	0.00 8643 303
ma rke r10	k__Bacteria|p__Fusobacteriota	D	2.61 2866 035	0.04 4421 207	0.04 4421 207
ma rke r11	k__Bacteria|p__Fusobacteriota|c__Fusobacteriia	D	2.61 2866 035	0.04 4421 207	0.04 4421 207
ma rke	k__Bacteria|p__Fusobacteriota|c__Fusobacteriia|o__Fusobacteri ales	D	2.61 2866	0.04 4421	0.04 4421

r12			035	207	207
ma<br>rke<br>r13	k__Bacteria|p__Proteobacteria|c__Gammaproteobacteria|o__Ent<br>erobacterales|f__Pasteurellaceae|g__Haemophilus|s__Haemophil<br>us_influenzae	D	2.56<br>1148<br>205	0.00<br>1445<br>257	0.00<br>1445<br>257
ma<br>rke<br>r14	k__Bacteria|p__Bacteroidota|c__Bacteroidia|o__Bacteroidales|f_<br>_Porphyromonadaceae	D	2.50<br>8938<br>545	0.01<br>0069<br>171	0.01<br>0069<br>171
ma<br>rke<br>r15	k__Bacteria|p__Bacteroidota|c__Bacteroidia|o__Bacteroidales|f_<br>_Porphyromonadaceae|g__Porphyromonas	D	2.50<br>8938<br>545	0.01<br>0069<br>171	0.01<br>0069<br>171
ma<br>rke<br>r16	k__Bacteria|p__Proteobacteria|c__Gammaproteobacteria|o__Ent<br>erobacterales|f__Pasteurellaceae|g__Actinobacillus	D	2.24<br>7406<br>551	0.00<br>0770<br>282	0.00<br>0770<br>282
ma<br>rke<br>r17	k__Bacteria|p__Proteobacteria|c__Gammaproteobacteria|o__Ent<br>erobacterales|f__Pasteurellaceae|g__Actinobacillus|s__Haemoph<br>ilus_parahaemolyticus	D	2.24<br>7323<br>273	0.00<br>0780<br>794	0.00<br>0780<br>794
ma<br>rke<br>r18	k__Bacteria|p__Patescibacteria|c__Gracilibacteria|o__Abscondit<br>abacteriales_(SR1)	D	2.23<br>4310<br>286	0.03<br>5570<br>577	0.03<br>5570<br>577
ma<br>rke<br>r19	k__Bacteria|p__Patescibacteria|c__Gracilibacteria|o__Abscondit<br>abacteriales_(SR1)|f__Absconditabacteriales_(SR1)	D	2.23<br>4310<br>286	0.03<br>5570<br>577	0.03<br>5570<br>577
ma<br>rke<br>r20	k__Bacteria|p__Patescibacteria|c__Gracilibacteria|o__Abscondit<br>abacteriales_(SR1)|f__Absconditabacteriales_(SR1)|g__Abscond<br>itabacteriales_(SR1)	D	2.23<br>4310<br>286	0.03<br>5570<br>577	0.03<br>5570<br>577
ma<br>rke<br>r21	k__Bacteria|p__Patescibacteria|c__Gracilibacteria|o__Abscondit<br>abacteriales_(SR1)|f__Absconditabacteriales_(SR1)|g__Abscond<br>itabacteriales_(SR1)|s__SR1_bacterium	D	2.23<br>4310<br>286	0.03<br>5570<br>577	0.03<br>5570<br>577
ma<br>rke<br>r22	k__Bacteria|p__Bacteroidota|c__Bacteroidia|o__Bacteroidales|f_<br>_Porphyromonadaceae|g__Porphyromonas|s__Porphyromonas_p<br>asteri	D	2.21<br>4392<br>719	0.04<br>1346<br>355	0.04<br>1346<br>355
ma<br>rke	k__Bacteria|p__Bacteroidota|c__Bacteroidia|o__Bacteroidales|f_<br>_Porphyromonadaceae|g__Porphyromonas|s__Porphyromonas_u	D	2.04<br>0229	0.00<br>0893	0.00<br>0893

r23	nidentified		977	48	48
marker24	k__Bacteria|p__Bacteroidota|c__Bacteroidia|o__Bacteroidales|f__Prevotellaceae|g__Prevotella_7|s__Prevotella_jejuni	H	2.775505587	0.012937694	0.012937694
marker25	k__Bacteria|p__Firmicutes|c__Negativicutes	H	2.763416685	0.022409915	0.022409915
marker26	k__Bacteria|p__Firmicutes|c__Negativicutes|o__Veillonellales-Selenomonadales	H	2.763198794	0.022520309	0.022520309
marker27	k__Bacteria|p__Firmicutes|c__Negativicutes|o__Veillonellales-Selenomonadales|f__Veillonellaceae	H	2.753433436	0.027736226	0.027736226
marker28	k__Bacteria|p__Bacteroidota|c__Bacteroidia|o__Bacteroidales|f__Prevotellaceae|g__Prevotella_7|s__Prevotella_denticola	H	2.022424148	0.017904868	0.017904868

Supplementary Table 6. Analysis of difference in saliva between adenoma (group E) and CRC (group F).

	feature	enrich_group	ef_lda	pvalue	padj
marker1	k__Bacteria|p__Proteobacteria|c__Gammaproteobacteria|o__Enterobacterales|f__Pasteurellaceae|g__Haemophilus	E	3.066152683	0.037243333	0.037243333
marker2	k__Bacteria|p__Proteobacteria|c__Gammaproteobacteria|o__Enterobacterales|f__Pasteurellaceae|g__Haemophilus|s__Haemophilus_influenzae	E	2.552402224	0.003086455	0.003086455
marker3	k__Bacteria|p__Bacteroidota|c__Bacteroidia|o__Bacteroidales|f__Porphyromonadaceae	E	2.455044293	0.012668092	0.012668092

marker 4	k__Bacteria|p__Bacteroidota|c__Bacteroidia|o__Bacteroidales|f__Porphyromonadaceae|g__Porphyromonas	E	2.455044293	0.012668092	0.012668092
marker 5	k__Bacteria|p__Bacteroidota|c__Bacteroidia|o__Bacteroidales|f__Prevotellaceae|g__Alloprevotella|s__Alloprevotella_uncultured_Bacteroidetes	E	2.352478816	0.022081551	0.022081551
marker 6	k__Bacteria|p__Spirochaetota	E	2.196464589	0.00431475	0.00431475
marker 7	k__Bacteria|p__Spirochaetota|c__Spirochaetia	E	2.196464589	0.00431475	0.00431475
marker 8	k__Bacteria|p__Spirochaetota|c__Spirochaetia|o__Spirochaetales	E	2.196464589	0.00431475	0.00431475
marker 9	k__Bacteria|p__Spirochaetota|c__Spirochaetia|o__Spirochaetales|f__Spirochaetaceae	E	2.196464589	0.00431475	0.00431475
marker 10	k__Bacteria|p__Spirochaetota|c__Spirochaetia|o__Spirochaetales|f__Spirochaetaceae|g__Treponema	E	2.195584521	0.00392497	0.00392497
marker 11	k__Bacteria|p__Bacteroidota|c__Bacteroidia|o__Bacteroidales|f__Prevotellaceae|g__Prevotella|s__Prevotella_nanceiensis	E	2.158651816	0.004656933	0.004656933
marker 12	k__Bacteria|p__Bacteroidota|c__Bacteroidia|o__Bacteroidales|f__Porphyromonadaceae|g__Porphyromonas|s__Porphyromonas_unidentified	E	2.130010642	0.01342173	0.01342173
marker 13	k__Bacteria|p__Proteobacteria|c__Gammaproteobacteria|o__Enterobacterales|f__Pasteurellaceae|g__Actinobacillus|s__Haemophilus_parahaemolyticus	E	2.07939129	0.030497734	0.030497734
marker 14	k__Bacteria|p__Proteobacteria|c__Gammaproteobacteria|o__Enterobacterales|f__Pasteurellaceae|g__Actinobacillus	E	2.079164752	0.029227115	0.029227115

marker15	k__Bacteria|p__Firmicutes|c__Clostridia	E	2.010655633	0.004140095	0.004140095
marker16	k__Bacteria|p__Bacteroidota|c__Bacteroidia|o__Flavobacteriales|f__Flavobacteriaceae|g__Capnocytophaga	H	2.193624687	0.025561195	0.025561195
marker17	k__Bacteria|p__Bacteroidota|c__Bacteroidia|o__Flavobacteriales|f__Flavobacteriaceae	H	2.184212217	0.040423228	0.040423228

Supplementary Table 7. Analysis of difference in feces between adenoma group and early CRC group.

	feature	enrich_group	ef_lda	pvalue	padj
marker1	k__Bacteria|p__Firmicutes|c__Clostridia|o__Lachnospirales|f__Lachnospiraceae|g__[Ruminococcus]_torques_group|s__[Ruminococcus]_torques_group_	B	4.088313177	0.00093944	0.00093944
marker2	k__Bacteria|p__Firmicutes|c__Clostridia|o__Lachnospirales|f__Lachnospiraceae|g__[Ruminococcus]_torques_group	B	4.088285979	0.00093944	0.00093944
marker3	k__Bacteria|p__Firmicutes|c__Bacilli|o__Lactobacillales|f__Streptococcaceae|g__Streptococcus|s__Streptococcus_	B	3.629968791	0.002684483	0.002684483
marker4	k__Bacteria|p__Proteobacteria|c__Gammaproteobacteria|o__Enterobacterales|f__Pasteurellaceae|g__Haemophilus|s__Haemophilus_	B	3.609937713	0.008538504	0.008538504
ma	k__Bacteria|p__Firmicutes|c__Clostridia|o__Lachnospirales|f__L	B	3.43	0.00	0.00

rke r5	achnospiraceae|g__Dorea		021 854 4	089 295 4	089 295 4
ma rke r6	k__Bacteria|p__Firmicutes|c__Clostridia|o__Lachnospirales|f__Lachnospiraceae|g__[Eubacterium]_hallii_group	B	3.39 052 253 2	0.00 882 794 9	0.00 882 794 9
ma rke r7	k__Bacteria|p__Firmicutes|c__Clostridia|o__Lachnospirales|f__Lachnospiraceae|g__[Eubacterium]_hallii_group|s__[Eubacterium]_hallii_group_	B	3.39 052 253 2	0.00 882 794 9	0.00 882 794 9
ma rke r8	k__Bacteria|p__Firmicutes|c__Clostridia|o__Lachnospirales|f__Lachnospiraceae|g__Dorea|s__uncultured_Dorea	B	3.13 628 825 2	0.00 045 377	0.00 045 377
ma rke r9	k__Bacteria|p__Actinobacteriota|c__Coriobacteriia|o__Coriobacteriales|f__Eggerthellaceae|g__Eggerthella|s__Eggerthella_uncultured_bacterium	B	2.44 710 370 5	0.00 329 019 6	0.00 329 019 6
ma rke r10	k__Bacteria|p__Actinobacteriota|c__Coriobacteriia|o__Coriobacteriales|f__Eggerthellaceae|g__Eggerthella	B	2.44 710 370 5	0.00 329 019 6	0.00 329 019 6
ma rke r11	k__Bacteria|p__Firmicutes|c__Negativicutes|o__Veillonellales-Selenomonadales|f__Veillonellaceae|g__Megasphaera|s__Megasphaera_elsdenii	Stag e I-II	3.51 646 029 9	0.00 724 081 2	0.00 724 081 2
ma rke r12	k__Bacteria|p__Bacteroidota|c__Bacteroidia|o__Bacteroidales|f__Rikenellaceae|g__Alistipes|s__Alistipes_shahii	Stag e I-II	3.21 833 529 7	0.00 278 029 6	0.00 278 029 6
ma rke r13	k__Bacteria|p__Actinobacteriota|c__Actinobacteria|o__Actinomycetales	Stag e I-II	3.11 431 859	0.00 018 119 7	0.00 018 119 7

marker14	k__Bacteria|p__Actinobacteriota|c__Actinobacteria|o__Actinomycetales|f__Actinomycetaceae	Stage I-II	3.112615109	0.000181197	0.000181197
marker15	k__Bacteria|p__Actinobacteriota|c__Actinobacteria|o__Actinomycetales|f__Actinomycetaceae|g__Actinomyces	Stage I-II	3.10879309	0.000192017	0.000192017
marker16	k__Bacteria|p__Actinobacteriota|c__Actinobacteria|o__Actinomycetales|f__Actinomycetaceae|g__Actinomyces|s__Schaalia_odontolytica	Stage I-II	3.053279823	0.000154871	0.000154871
marker17	k__Bacteria|p__Firmicutes|c__Clostridia|o__Oscillospirales|f__Ruminococcaceae|g__Ruminococcus|s__Ruminococcus_bicirculans	Stage I-II	3.038844319	0.002770847	0.002770847
marker18	k__Bacteria|p__Bacteroidota|c__Bacteroidia|o__Bacteroidales|f__Porphyromonadaceae|g__Porphyromonas|s__Porphyromonas_uenonis	Stage I-II	3.015628676	0.0002529	0.0002529
marker19	k__Bacteria|p__Firmicutes|c__Clostridia|o__Lachnospirales|f__Lachnospiraceae|g__[Eubacterium]_ventriosum_group|s__[Eubacterium]_ventriosum_group_uncultured_Eubacterium	Stage I-II	2.882616822	0.00342937	0.00342937
marker20	k__Bacteria|p__Firmicutes|c__Clostridia|o__Peptostreptococcales-Tissierellales|f__Peptostreptococcaceae|g__Peptostreptococcus|s__Peptostreptococcus_uncultured_organism	Stage I-II	2.687044628	0.007147597	0.007147597
marker21	k__Bacteria|p__Firmicutes|c__Clostridia|o__Peptostreptococcales-Tissierellales|f__Peptostreptococcaceae|g__Peptostreptococcus	Stage I-II	2.687044628	0.007147597	0.007147597
marker22	k__Bacteria|p__Firmicutes|c__Negativicutes|o__Veillonellales-Selenomonadales|f__Veillonellaceae|g__Dialister|s__Dialister_pneumosintes	Stage I-II	2.625977387	0.004979118	0.004979118

marker23	k__Bacteria|p__Firmicutes|c__Negativicutes|o__Acidaminococcales|f__Acidaminococcaceae|g__Phascolarctobacterium|s__Phascolarctobacterium_uncultured_Firmicutes	Stage I-II	2.396167107	0.000541846	0.000541846
marker24	k__Bacteria|p__Bacteroidota|c__Bacteroidia|o__Bacteroidales|f__Prevotellaceae|g__Prevotella|s__Prevotella_intermedia	Stage I-II	2.304890152	0.005950655	0.005950655
marker25	k__Bacteria|p__Firmicutes|c__Negativicutes|o__Acidaminococcales|f__Acidaminococcaceae|g__Acidaminococcus|s__Acidaminococcus_sp.	Stage I-II	2.215331928	0.001888434	0.001888434

Supplementary Table 8. Analysis of difference in feces between early CRC group and late stage group.

	feature	enrich_group	ef_lda	pvalue	padj
marker1	k__Bacteria|p__Firmicutes|c__Clostridia|o__Lachnospirales	Stage I-II	4.415238711	0.098730276	0.098730276
marker2	k__Bacteria|p__Firmicutes|c__Clostridia|o__Lachnospirales|f__Lachnospiraceae	Stage I-II	4.415121285	0.098730276	0.098730276
marker3	k__Bacteria|p__Firmicutes|c__Negativicutes	Stage I-II	4.365187354	0.022498971	0.022498971
marke	k__Bacteria|p__Firmicutes|c__Negativicutes|o__Veillonellales-Selenomonadales	Stage I-	4.33573775	0.07463758	0.07463758

<table>
<tr><td>r4</td><td></td><td>II</td><td>5</td><td>1</td><td>1</td></tr>
<tr><td>marker5</td><td>k__Bacteria|p__Firmicutes|c__Clostridia|o__Lachnospirales|f__Lachnospiraceae|g__Roseburia</td><td>Stage I-II</td><td>3.98559483</td><td>0.028626743</td><td>0.028626743</td></tr>
<tr><td>marker6</td><td>k__Bacteria|p__Firmicutes|c__Clostridia|o__Lachnospirales|f__Lachnospiraceae|g__Roseburia|s__Roseburia_uncultured_organism</td><td>Stage I-II</td><td>3.96336696</td><td>0.031108844</td><td>0.031108844</td></tr>
<tr><td>marker7</td><td>k__Bacteria|p__Firmicutes|c__Clostridia|o__Lachnospirales|f__Lachnospiraceae|g__Lachnospira</td><td>Stage I-II</td><td>3.606352008</td><td>0.009548783</td><td>0.009548783</td></tr>
<tr><td>marker8</td><td>k__Bacteria|p__Firmicutes|c__Clostridia|o__Lachnospirales|f__Lachnospiraceae|g__Lachnospira|s__Lachnospira_</td><td>Stage I-II</td><td>3.586098178</td><td>0.011183802</td><td>0.011183802</td></tr>
<tr><td>marker9</td><td>k__Bacteria|p__Firmicutes|c__Clostridia|o__Lachnospirales|f__Lachnospiraceae|g__Lachnoclostridium</td><td>Stage I-II</td><td>3.556981595</td><td>0.071996437</td><td>0.071996437</td></tr>
<tr><td>marker10</td><td>k__Bacteria|p__Firmicutes|c__Clostridia|o__Lachnospirales|f__Lachnospiraceae|g__[Eubacterium]_eligens_group</td><td>Stage I-II</td><td>3.538466501</td><td>0.028230094</td><td>0.028230094</td></tr>
<tr><td>marker11</td><td>k__Bacteria|p__Firmicutes|c__Clostridia|o__Lachnospirales|f__Lachnospiraceae|g__[Eubacterium]_eligens_group|s__[Eubacterium]_eligens_group_</td><td>Stage I-II</td><td>3.538466501</td><td>0.028230094</td><td>0.028230094</td></tr>
<tr><td>marker12</td><td>k__Bacteria|p__Firmicutes|c__Clostridia|o__Lachnospirales|f__Lachnospiraceae|g__Lachnoclostridium|s__Lachnoclostridium_</td><td>Stage I-II</td><td>3.4182427</td><td>0.047686481</td><td>0.047686481</td></tr>
<tr><td>marke</td><td>k__Bacteria|p__Firmicutes|c__Negativicutes|o__Acidaminococcales</td><td>Stage I-</td><td>3.41635376</td><td>0.09761537</td><td>0.09761537</td></tr>
</table>

r13		II	2	9	9
marker14	k__Bacteria|p__Firmicutes|c__Negativicutes|o__Acidaminococcales|f__Acidaminococcaceae	Stage I-II	3.416353762	0.097615379	0.097615379
marker15	k__Bacteria|p__Firmicutes|c__Negativicutes|o__Acidaminococcales|f__Acidaminococcaceae|g__Phascolarctobacterium|s__Phascolarctobacterium_uncultured_organism	Stage I-II	3.386503436	0.020929001	0.020929001
marker16	k__Bacteria|p__Firmicutes|c__Clostridia|o__Peptostreptococcales-Tissierellales|f__Peptostreptococcaceae|g__Romboutsia	Stage I-II	3.213145587	0.06613067	0.06613067
marker17	k__Bacteria|p__Firmicutes|c__Clostridia|o__Peptostreptococcales-Tissierellales|f__Peptostreptococcaceae|g__Romboutsia|s__Romboutsia_	Stage I-II	3.213145587	0.06613067	0.06613067
marker18	k__Bacteria|p__Proteobacteria|c__Gammaproteobacteria|o__Enterobacterales|f__Pasteurellaceae	Stage I-II	3.012227872	0.099855315	0.099855315
marker19	k__Bacteria|p__Firmicutes|c__Clostridia|o__Lachnospirales|f__Lachnospiraceae|g__Lachnospiraceae_ND3007_group	Stage I-II	2.742267869	0.027068435	0.027068435
marker20	k__Bacteria|p__Firmicutes|c__Clostridia|o__Lachnospirales|f__Lachnospiraceae|g__Lachnospiraceae_ND3007_group|s__Lachnospiraceae_ND3007_group_metagenome	Stage I-II	2.741405786	0.028626743	0.028626743
marker21	k__Bacteria|p__Firmicutes|c__Clostridia|o__Oscillospirales|f__Ruminococcaceae|g__Paludicola	Stage I-II	2.180176285	0.091151233	0.091151233
marke	k__Bacteria|p__Proteobacteria|c__Gammaproteobacteria|o__Ente	Stage I-	2.03319	0.08931	0.08931

r22	robacterales|f__Yersiniaceae	II	2122	7742	7742
marker23	k__Bacteria|p__Proteobacteria|c__Gammaproteobacteria|o__Enterobacterales|f__Yersiniaceae|g__Serratia|s__Serratia_	Stage I-II	2.026637518	0.089317742	0.089317742
marker24	k__Bacteria|p__Proteobacteria|c__Gammaproteobacteria|o__Enterobacterales|f__Yersiniaceae|g__Serratia	Stage I-II	2.024089318	0.089317742	0.089317742
marker25	k__Bacteria|p__Actinobacteriota|c__Actinobacteria|o__Bifidobacteriales|f__Bifidobacteriaceae|g__Bifidobacterium|s__Bifidobacterium_longum	Stage III-IV	3.410178097	0.083985622	0.083985622
marker26	k__Bacteria|p__Firmicutes|c__Clostridia|o__Peptostreptococcales-Tissierellales|f__Peptostreptococcaceae|g__Peptostreptococcus	Stage III-IV	2.814008562	0.078654758	0.078654758
marker27	k__Bacteria|p__Firmicutes|c__Clostridia|o__Peptostreptococcales-Tissierellales|f__Peptostreptococcaceae|g__Peptostreptococcus|s__Peptostreptococcus_uncultured_organism	Stage III-IV	2.814008562	0.078654758	0.078654758
marker28	k__Bacteria|p__Firmicutes|c__Clostridia|o__Oscillospirales|f__Oscillospiraceae|g__UCG-005|s__UCG-005_	Stage III-IV	2.442662733	0.08378377	0.08378377
marker29	k__Bacteria|p__Firmicutes|c__Clostridia|o__Oscillospirales|f__Oscillospiraceae|g__UCG-002|s__UCG-002_uncultured_rumen	Stage III-IV	2.426068654	0.078178396	0.078178396
marker30	k__Bacteria|p__Firmicutes|c__Clostridia|o__Oscillospirales|f__Ruminococcaceae|g__Incertae_Sedis|s__Incertae_Sedis_uncultured_organism	Stage III-IV	2.217699781	0.070617815	0.070617815
marke	k__Bacteria|p__Bacteroidota|c__Bacteroidia|o__Bacteroidales|f_	Stage	2.14834	0.02378	0.02378

r31	_Bacteroidaceae|g__Bacteroides|s__Bacteroides_acidifaciens	III-IV	335	0663	0663

Supplementary Table 9. Analysis of difference in saliva between adenoma group and early CRC group.

	feature	enrich_group	ef_lda	pvalue	padj
marker1	k__Bacteria|p__Proteobacteria|c__Gammaproteobacteria|o__Enterobacterales|f__Pasteurellaceae|g__Haemophilus|s__Haemophilus_influenzae	E	3.870665709	0.001171845	0.001171845
marker2	k__Bacteria|p__Patescibacteria|c__Gracilibacteria|o__Absconditabacteriales_(SR1)	E	3.517734663	0.006855996	0.006855996
marker3	k__Bacteria|p__Patescibacteria|c__Gracilibacteria|o__Absconditabacteriales_(SR1)|f__Absconditabacteriales_(SR1)	E	3.517734663	0.006855996	0.006855996
marker4	k__Bacteria|p__Patescibacteria|c__Gracilibacteria|o__Absconditabacteriales_(SR1)|f__Absconditabacteriales_(SR1)|g__Absconditabacteriales_(SR1)	E	3.517734663	0.006855996	0.006855996
marker5	k__Bacteria|p__Patescibacteria|c__Gracilibacteria|o__Absconditabacteriales_(SR1)|f__Absconditabacteriales_(SR1)|g__Absconditabacteriales_(SR1)|s__SR1_bacterium	E	3.517734663	0.006855996	0.006855996
marker6	k__Bacteria|p__Bacteroidota|c__Bacteroidia|o__Bacteroidales|f__Prevotellaceae|g__Prevotella|s__Prevotella_nanceiensis	E	3.474495288	0.007215384	0.007215384
marker7	k__Bacteria|p__Proteobacteria|c__Gammaproteobacteria|o__Enterobacterales|f__Pasteurellaceae|g__Actinobacillus|s__Haemophilus_parahaemolyticus	E	3.221997558	0.005197257	0.005197257
marke	k__Bacteria|p__Proteobacteria|c__Gammaproteobacteria|o__Ent	E	3.221946	0.005017	0.005017

r8	erobacterales|f__Pasteurellaceae|g__Actinobacillus		562	706	706
ma rke r9	k__Bacteria|p__Bacteroidota|c__Bacteroidia|o__Bacteroidales|f__Prevotellaceae|g__Prevotella|s__Prevotella_aurantiaca	E	3.20 7682 023	0.00 1355 179	0.00 1355 179
ma rke r10	k__Bacteria|p__Firmicutes|c__Bacilli|o__Acholeplasmatales	E	3.02 5813 835	0.00 0320 89	0.00 0320 89
ma rke r11	k__Bacteria|p__Firmicutes|c__Bacilli|o__Acholeplasmatales|f__Acholeplasmataceae|g__Acholeplasma	E	3.02 5473 504	0.00 0320 89	0.00 0320 89
ma rke r12	k__Bacteria|p__Firmicutes|c__Bacilli|o__Acholeplasmatales|f__Acholeplasmataceae	E	3.02 5291 968	0.00 0320 89	0.00 0320 89
ma rke r13	k__Bacteria|p__Spirochaetota|c__Spirochaetia|o__Spirochaetales|f__Spirochaetaceae|g__Treponema|s__Treponema_sp.	E	2.53 9493 068	0.00 1859 488	0.00 1859 488
ma rke r14	k__Bacteria|p__Proteobacteria|c__Gammaproteobacteria|o__Burkholderiales|f__Neisseriaceae|g__Neisseria|s__Neisseria_oralis	E	2.53 6128 45	0.00 8958 247	0.00 8958 247
ma rke r15	k__Bacteria|p__Firmicutes|c__Clostridia|o__Lachnospirales|f__Lachnospiraceae|g__Lachnoanaerobaculum|s__Lachnoanaerobaculum_uncultured_bacterium	E	2.48 8503 787	0.00 1070 998	0.00 1070 998
ma rke r16	k__Bacteria|p__Firmicutes|c__Clostridia|o__Lachnospirales|f__Lachnospiraceae|g__Lachnoanaerobaculum	E	2.48 8503 787	0.00 1070 998	0.00 1070 998
ma rke r17	k__Bacteria|p__Actinobacteriota|c__Coriobacteriia	Stag e I-II	3.25 3729 916	0.00 3113 931	0.00 3113 931
ma rke r18	k__Bacteria|p__Actinobacteriota|c__Coriobacteriia|o__Coriobacteriales	Stag e I-II	3.25 3729 916	0.00 3113 931	0.00 3113 931
ma rke	k__Bacteria|p__Actinobacteriota|c__Coriobacteriia|o__Coriobacteriales|f__Atopobiaceae	Stag e I-II	3.23 8462	0.00 6292	0.00 6292

r19			887	627	627
marker20	k__Bacteria|p__Firmicutes|c__Bacilli|o__Lactobacillales|f__Lactobacillaceae	Stage I-II	3.220360419	0.009320958	0.009320958
marker21	k__Bacteria|p__Actinobacteriota|c__Coriobacteriia|o__Coriobacteriales|f__Atopobiaceae|g__Atopobium	Stage I-II	3.209457437	0.008397167	0.008397167
marker22	k__Bacteria|p__Actinobacteriota|c__Coriobacteriia|o__Coriobacteriales|f__Atopobiaceae|g__Atopobium|s__Atopobium_uncultured_bacterium	Stage I-II	3.207884352	0.008827949	0.008827949
marker23	k__Bacteria|p__Firmicutes|c__Bacilli|o__Lactobacillales|f__Streptococcaceae|g__Streptococcus|s__Streptococcus_anginosus	Stage I-II	2.874294486	0.000574712	0.000574712
marker24	k__Bacteria|p__Firmicutes|c__Bacilli|o__Lactobacillales|f__Lactobacillaceae|g__Limosilactobacillus	Stage I-II	2.716220432	9.48E-05	9.48E-05
marker25	k__Bacteria|p__Firmicutes|c__Bacilli|o__Lactobacillales|f__Lactobacillaceae|g__Lactobacillus|s__Lactobacillus_	Stage I-II	2.676983334	0.003507925	0.003507925
marker26	k__Bacteria|p__Firmicutes|c__Bacilli|o__Lactobacillales|f__Lactobacillaceae|g__Ligilactobacillus	Stage I-II	2.157512221	0.003735063	0.003735063
marker27	k__Bacteria|p__Firmicutes|c__Bacilli|o__Lactobacillales|f__Lactobacillaceae|g__Ligilactobacillus|s__Lactobacillus_salivarius	Stage I-II	2.057006275	0.001565471	0.001565471
marker28	k__Bacteria|p__Bacteroidota|c__Bacteroidia|o__Bacteroidales|f__Prevotellaceae|g__Prevotella_7|s__Prevotella_7_uncultured_Prevotella	Stage I-II	2.05567459	0.009710678	0.009710678

Supplementary Table 10. Analysis of difference in saliva between early CRC group and late stage group.

	feature	enrich_group	ef_lda	pvalue	padj
marker1	k__Bacteria|p__Bacteroidota|c__Bacteroidia|o__Bacteroidales|f__Prevotellaceae|g__Prevotella_7|s__Prevotella_jejuni	Stage I-II	4.058366765	0.090090195	0.090090195
marker2	k__Bacteria|p__Proteobacteria|c__Gammaproteobacteria|o__Enterobacterales|f__Pasteurellaceae|g__Actinobacillus	Stage I-II	3.523081992	0.092190993	0.092190993
marker3	k__Bacteria|p__Proteobacteria|c__Gammaproteobacteria|o__Enterobacterales|f__Pasteurellaceae|g__Actinobacillus|s__Haemophilus_parahaemolyticus	Stage I-II	3.522565247	0.092190993	0.092190993
marker4	k__Bacteria|p__Actinobacteriota|c__Coriobacteriia|o__Coriobacteriales|f__Atopobiaceae	Stage I-II	3.377585635	0.01357439	0.01357439
marker5	k__Bacteria|p__Actinobacteriota|c__Coriobacteriia	Stage I-II	3.372258435	0.018615408	0.018615408
marker6	k__Bacteria|p__Actinobacteriota|c__Coriobacteriia|o__Coriobacteriales	Stage I-II	3.372258435	0.018615408	0.018615408
marker7	k__Bacteria|p__Actinobacteriota|c__Coriobacteriia|o__Coriobacteriales|f__Atopobiaceae|g__Atopobium	Stage I-II	3.364715755	0.016282946	0.016282946
marker8	k__Bacteria|p__Actinobacteriota|c__Coriobacteriia|o__Coriobacteriales|f__Atopobiaceae|g__Atopobium|s__Atopobium_uncultured_bacterium	Stage I-II	3.362853319	0.017414754	0.017414754
marker9	k__Bacteria|p__Proteobacteria|c__Gammaproteobacteria|o__Pseudomonadales|f__Moraxellaceae	Stage I-II	3.300092405	0.063516878	0.063516878
marker10	k__Bacteria|p__Spirochaetota|c__Spirochaetia|o__Spirochaetales|f__Spirochaetaceae|g__Treponema|s__Treponema_medium	Stage I-II	3.281451479	0.093032788	0.093032788

marker 11	k__Bacteria|p__Bacteroidota|c__Bacteroidia|o__Bacteroidales|f__Porphyromonadaceae|g__Porphyromonas|s__Porphyromonas_gingivalis	Stage I-II	3.239410034	0.070278574	0.070278574
marker 12	k__Bacteria|p__Bacteroidota|c__Bacteroidia|o__Bacteroidales|f__Prevotellaceae|g__Prevotella_7|s__Prevotella_7_Prevotella_sp.	Stage I-II	2.890464032	0.042405517	0.042405517
marker 13	k__Bacteria|p__Bacteroidota|c__Bacteroidia|o__Bacteroidales|f__Prevotellaceae|g__Prevotella_7|s__Prevotella_7_	Stage I-II	2.808499888	0.054855862	0.054855862
marker 14	k__Bacteria|p__Firmicutes|c__Bacilli|o__Lactobacillales|f__Lactobacillaceae|g__Lactobacillus|s__Lactobacillus_gasseri	Stage I-II	2.773694087	0.059398446	0.059398446
marker 15	k__Bacteria|p__Spirochaetota|c__Spirochaetia|o__Spirochaetales|f__Spirochaetaceae|g__Treponema|s__Treponema_	Stage I-II	2.521104032	0.084077237	0.084077237
marker 16	k__Bacteria|p__Firmicutes|c__Clostridia|o__Lachnospirales|f__Lachnospiraceae|g__Shuttleworthia	Stage I-II	2.153924545	0.026776965	0.026776965
marker 17	k__Bacteria|p__Firmicutes|c__Clostridia|o__Lachnospirales|f__Lachnospiraceae|g__Shuttleworthia|s__Shuttleworthia_satelles	Stage I-II	2.153869818	0.026776965	0.026776965
marker 18	k__Bacteria|p__Bacteroidota|c__Bacteroidia|o__Bacteroidales|f__Rikenellaceae|g__Rikenellaceae_RC9_gut_group	Stage I-II	2.026363475	0.087827064	0.087827064
marker 19	k__Bacteria|p__Bacteroidota|c__Bacteroidia|o__Bacteroidales|f__Prevotellaceae|g__Alloprevotella	Stage III-IV	4.143161607	0.064528989	0.064528989
marker 20	k__Bacteria|p__Bacteroidota|c__Bacteroidia|o__Flavobacteriales|f__Flavobacteriaceae	Stage III-IV	3.618701054	0.020626726	0.020626726
marker 21	k__Bacteria|p__Bacteroidota|c__Bacteroidia|o__Flavobacteriales|f__Flavobacteriaceae|g__Capnocytophaga	Stage III-IV	3.618701054	0.020626726	0.020626726

marker 22	k__Bacteria|p__Bacteroidota|c__Bacteroidia|o__Flavobacteriales	Stage III-IV	3.494932686	0.077357349	0.077357349
marker 23	k__Bacteria|p__Proteobacteria|c__Gammaproteobacteria|o__Enterobacterales|f__Enterobacteriaceae|g__Escherichia-Shigella|s__Escherichia-Shigella_	Stage III-IV	3.197512184	0.06570234	0.06570234
marker 24	k__Bacteria|p__Bacteroidota|c__Bacteroidia|o__Flavobacteriales|f__Flavobacteriaceae|g__Capnocytophaga|s__Capnocytophaga_gingivalis	Stage III-IV	3.193454098	0.003711879	0.003711879
marker 25	k__Bacteria|p__Proteobacteria|c__Gammaproteobacteria|o__Enterobacterales|f__Enterobacteriaceae|g__Escherichia-Shigella	Stage III-IV	3.192920073	0.06570234	0.06570234
marker 26	k__Bacteria|p__Firmicutes|c__Bacilli|o__Lactobacillales|f__Enterococcaceae|g__Enterococcus|s__Enterococcus_	Stage III-IV	3.017838517	0.066541991	0.066541991
marker 27	k__Bacteria|p__Firmicutes|c__Bacilli|o__Lactobacillales|f__Enterococcaceae|g__Enterococcus	Stage III-IV	3.014841638	0.066541991	0.066541991
marker 28	k__Bacteria|p__Firmicutes|c__Bacilli|o__Lactobacillales|f__Enterococcaceae	Stage III-IV	3.013171584	0.066541991	0.066541991
marker 29	k__Bacteria|p__Proteobacteria|c__Gammaproteobacteria|o__Burkholderiales|f__Comamonadaceae	Stage III-IV	2.835590836	0.033694526	0.033694526
marker 30	k__Bacteria|p__Proteobacteria|c__Gammaproteobacteria|o__Burkholderiales|f__Comamonadaceae|g__Comamonas|s__Ottowia_sp.	Stage III-IV	2.829311353	0.01470889	0.01470889
marker 31	k__Bacteria|p__Proteobacteria|c__Gammaproteobacteria|o__Burkholderiales|f__Comamonadaceae|g__Comamonas	Stage III-IV	2.825468254	0.020530694	0.020530694
marker 32	k__Bacteria|p__Bacteroidota|c__Bacteroidia|o__Flavobacteriales|f__Flavobacteriaceae|g__Capnocytophaga|s__uncultured_Capnocytophaga	Stage III-IV	2.562481092	0.082508941	0.082508941

marker 33	k__Bacteria|p__Bacteroidota|c__Bacteroidia|o__Bacteroidales|f__Bacteroidaceae|g__Bacteroides|s__Bacteroides_uniformis	Stage III-IV	2.522070163	0.071925932	0.071925932
marker 34	k__Bacteria|p__Proteobacteria|c__Alphaproteobacteria|o__Rickettsiales|f__Mitochondria|g__Mitochondria	Stage III-IV	2.496123776	0.068571044	0.068571044
marker 35	k__Bacteria|p__Proteobacteria|c__Alphaproteobacteria|o__Rickettsiales|f__Mitochondria|g__Mitochondria|s__Mitochondria_	Stage III-IV	2.495968364	0.068571044	0.068571044
marker 36	k__Bacteria|p__Proteobacteria|c__Alphaproteobacteria|o__Rickettsiales|f__Mitochondria	Stage III-IV	2.493699462	0.068571044	0.068571044
marker 37	k__Bacteria|p__Firmicutes|c__Clostridia|o__Lachnospirales|f__Lachnospiraceae|g__Lachnospiraceae_NK4A136_group	Stage III-IV	2.435957784	0.035073102	0.035073102
marker 38	k__Bacteria|p__Firmicutes|c__Clostridia|o__Clostridiales|f__Clostridiaceae|g__Clostridium_sensu_stricto_1|s__Clostridium_sensu_stricto_1_	Stage III-IV	2.119847701	0.040560684	0.040560684
marker 39	k__Bacteria|p__Desulfobacterota|c__Desulfovibrionia|o__Desulfovibrionales|f__Desulfovibrionaceae	Stage III-IV	2.098992171	0.047926674	0.047926674
marker 40	k__Bacteria|p__Firmicutes|c__Clostridia|o__Oscillospirales|f__Oscillospiraceae|g__UCG-005	Stage III-IV	2.087762682	0.069090211	0.069090211
marker 41	k__Bacteria|p__Firmicutes|c__Clostridia|o__Oscillospirales|f__Ruminococcaceae|g__Ruminococcus	Stage III-IV	2.07201916	0.077138124	0.077138124

Supplementary Table 11. Screening the OTU shared by normal, adenoma and CRC in feces and saliva.

	Kingdom	Phylum	Class	Order	Family	Genus	Species
ASV1	Bacteria	Firmicutes	Bacilli	Lactobacillales	Streptococcaceae	Streptococcus	
ASV2	Bacteria	Proteobacteria	Gammaproteobacteria	Enterobacterales	Enterobacteriaceae	Escherichia-Shigella	
ASV3	Bacteria	Proteobacteria	Gammaproteobacteria	Enterobacterales	Pasteurellaceae	Haemophilus	
ASV4	Bacteria	Firmicutes	Clostridia	Oscillospirales	Ruminococcaceae	Faecalibacterium	
ASV9	Bacteria	Fusobacteriota	Fusobacteriia	Fusobacteriales	Fusobacteriaceae	Fusobacterium	Fusobacterium_periodonticum
ASV23	Bacteria	Proteobacteria	Gammaproteobacteria	Pseudomonadales	Pseudomonadaceae	Pseudomonas	
ASV29	Bacteria	Firmicutes	Bacilli	Lactobacillales	Streptococcaceae	Streptococcus	Streptococcus_salivarius
ASV30	Bacteria	Firmicutes	Negativicutes	Veillonellales-Selenomonadales	Veillonellaceae	Veillonella	
ASV33	Bacteria	Firmicutes	Bacilli	Lactobacillales	Carnobacteriaceae	Granulicatella	
ASV66	Bacteria	Firmicutes	Negativicutes	Veillonellales-Selenomonadales	Veillonellaceae	Dialister	
ASV70	Bacteria	Firmicutes	Bacilli	Staphylococcales	Gemellaceae	Gemella	
ASV79	Bacteria	Firmicutes	Clostridia	Peptostreptococcales-Tissierellales	Peptostreptococcaceae	Peptostreptococcus	uncultured_organism
AS	Bact	Actinob	Actinobact	Micrococcales	Micrococc	Rothia	

V90	eria	acteriota	eria		aceae		
ASV104	Bacteria	Firmicutes	Clostridia	Peptostreptococcales-Tissierellales	Family_XI	Parvimonas	
ASV126	Bacteria	Firmicutes	Bacilli	Lactobacillales	Streptococcaceae	Streptococcus	Streptococcus_anginosus
ASV131	Bacteria	Actinobacteriota	Actinobacteria	Actinomycetales	Actinomycetaceae	Actinomyces	Schaalia_odontolytica
ASV171	Bacteria	Firmicutes	Negativicutes	Veillonellales-Selenomonadales	Veillonellaceae	Dialister	Dialister_pneumosintes

Supplementary Table 12. Analysis of the difference of KEGG pathways between normal group and adenoma fecal group.

	var	estimate	conf.low	conf.high	p.value	Group
1	Protein families: genetic information processing	0.204868251	-0.021061786	0.430798288	0.0386	A
2	Translation	0.069135519	-0.017997953	0.15626899	0.0377	A
3	Replication and repair	0.045550799	-0.019098776	0.110200374	0.0414	A
4	Protein families: metabolism	0.03287826	0.000443959	0.065312561	0.0242	A

5	sorting and degradation	0.019443489	-0.00147102	0.040357997	0.0135	A
6	Folding	0.019443489	-0.00147102	0.040357997	0.0135	A
7	Transcription	0.00476215	0.000400443	0.009123857	0.0108	A
8	Not included in regular maps	0.002180143	0.000319858	0.004040427	0.0322	A
9	Endocrine and metabolic disease	0.002074689	-0.000688837	0.004838215	0.0455	A
10	Cellular community - eukaryotes	-6.09E-06	-1.92E-05	7.00E-06	0.00229	B
11	Development and regeneration	-0.001509358	-0.002681132	-0.000337583	0.0199	B
12	Excretory system	-0.002191197	-0.004012814	-0.000369579	0.0132	B
13	Aging	-0.007606637	-0.015058767	-0.000154507	0.0426	B
14	Neurodegenerative disease	-0.013486394	-0.022797022	-0.004175765	0.00712	B
15	Lipid metabolism	-0.022433412	-0.036428302	-0.008438523	0.00277	B
16	Metabolism of other amino acids	-0.029209	-0.050512	-0.007905	0.0105	B

		098	93	266		
17	Signal transduction	-0.049674799	-0.114066858	0.01471726	0.0149	B
18	Unclassified: metabolism	-0.064266793	-0.13920524	0.010671655	0.0217	B

Supplementary Table 13. Analysis of the difference of KEGG pathways between normal group and colorectal cancer fecal group.

	var	estimate	conf.low	conf.high	p.value	Group
1	Unclassified: signaling and cellular processes	0.040071755	0.019400519	0.060742991	0.000212	A
2	sorting and degradation	0.021114516	-0.000631064	0.042860096	0.0197	A
3	Folding	0.021114516	-0.000631064	0.042860096	0.0197	A
4	Endocrine and metabolic disease	0.003102476	-1.88E-05	0.006223783	0.0336	A
5	Not included in regular maps	0.002289859	0.000546368	0.00403335	0.014	A
6	Cellular community - eukaryotes	-2.22E-05	-4.26E-05	-1.73E-06	5.83E-05	C
7	Infectious disease: viral	-0.000967	-0.002723	0.000788558	0.0453	C

		393	345			
8	Excretory system	-0.003592061	-0.005501643	-0.001682478	0.000252	C
9	Cancer: overview	-0.005140797	-0.009162228	-0.001119365	0.0117	C
10	Environmental adaptation	-0.006615725	-0.013266305	3.49E-05	0.0222	C
11	Digestive system	-0.010329487	-0.015779221	-0.004879753	8.52E-05	C
12	Neurodegenerative disease	-0.013426733	-0.020967423	-0.005886043	0.000243	C
13	Infectious disease: bacterial	-0.014329183	-0.025522725	-0.003135641	0.00798	C
14	Aging	-0.014925392	-0.022528951	-0.007321833	0.00025	C
15	Drug resistance: antimicrobial	-0.020389924	-0.039156462	-0.001623386	0.0292	C
16	Metabolism of other amino acids	-0.028025348	-0.047703049	-0.008347647	0.00423	C
17	Unclassified: metabolism	-0.083350575	-0.162578609	-0.004122541	0.018	C
18	Glycan biosynthesis and metabolism	-0.096543	-0.186384	-0.006703	0.0443	C

		773	265	281		

Supplementary Table 14. Analysis of the difference of KEGG pathways between adenoma and intestinal cancer fecal group.

	var	estimate	conf.low	conf.high	p.value	Group
1	Development and regeneration	0.001459396	0.000333619	0.002585173	0.0169	B
2	Digestive system	-0.006332471	-0.01184847	-0.000816471	0.0123	C

Supplementary Table 15. Analysis of the difference of KEGG pathways between normal and adenoma saliva groups.

	var	estimate	conf.low	conf.high	p.value	Group
1	Membrane transport	0.10985407	0.024932985	0.194775155	0.0184	D
2	Poorly characterized	0.052640346	0.020872825	0.084407867	0.000983	D
3	Unclassified: metabolism	0.03816654	0.014729155	0.061603925	0.000815	D
4	Unclassified: signaling and cellular processes	0.025858964	0.00917149	0.042546437	0.000783	D
5	Cancer: specific types	0.00446795	0.001143435	0.007792465	0.0104	D

6	Cardiovascular disease	0.002452126	-0.000121675	0.005025928	0.0315	D
7	Development and regeneration	0.001912846	0.00084435	0.002981343	0.000384	D
8	Transcription	-0.001640539	-0.003305901	2.48E-05	0.00581	E
9	Infectious disease: bacterial	-0.008598104	-0.015484826	-0.001711381	0.00685	E
10	Digestive system	-0.008882569	-0.015011542	-0.002753595	0.00966	E
11	Immune system	-0.010297791	-0.016950451	-0.003645132	0.0041	E
12	Transport and catabolism	-0.014613278	-0.024128596	-0.005097961	0.00941	E
13	Metabolism of terpenoids and polyketides	-0.017479411	-0.030885465	-0.004073358	0.012	E
14	Cell growth and death	-0.019697083	-0.03302586	-0.006368307	0.00674	E
15	Replication and repair	-0.026397745	-0.05211909	-0.000676399	0.0125	E
16	Nucleotide metabolism	-0.035363931	-0.058231178	-0.012496684	0.00359	E

17	Translation	-0.049405271	-0.085010753	-0.01379979	0.00108	E
18	Carbohydrate metabolism	-0.062774587	-0.109628492	-0.015920682	0.0208	E
19	Glycan biosynthesis and metabolism	-0.068556542	-0.115459133	-0.021653951	0.0188	E

Supplementary Table 16. Analysis of the difference of KEGG pathways between normal and colorectal cancer saliva groups.

	var	estimate	conf.low	conf.high	p.value	Group
1	Protein families: genetic information processing	0.157165581	-0.014900738	0.329231899	0.016	D
2	Membrane transport	0.096778195	0.003451711	0.190104679	0.0328	D
3	Poorly characterized	0.067310594	0.032480112	0.102141075	0.00025	D
4	Unclassified: metabolism	0.055049346	0.028921582	0.08117711	2.04E-05	D
5	sorting and degradation	0.034558609	0.019177291	0.049939927	3.72E-05	D
6	Folding	0.034558609	0.019177291	0.049939927	3.72E-05	D
7	Unclassified: signaling and cellular	0.031451	0.014548	0.048354	0.0001	D

	processes	6	63	571	38	
8	Cancer: specific types	0.002366268	-0.003741891	0.008474427	0.00218	D
9	Development and regeneration	0.002170783	0.000982639	0.003358927	0.000978	D
10	Cardiovascular disease	0.001608245	-0.002402509	0.005618998	0.0109	D
11	Endocrine and metabolic disease	0.001126335	-0.00082368	0.003076351	0.0449	D
12	Transcription	-0.000821105	-0.003232916	0.001590705	0.0148	H
13	Excretory system	-0.001233783	-0.002193216	-0.000274349	0.0275	H
14	Nervous system	-0.00515018	-0.008232264	-0.002068096	0.00369	H
15	Infectious disease: viral	-0.008001088	-0.018969588	0.002967412	0.00606	H
16	Immune system	-0.008003835	-0.014979533	-0.001028138	0.0193	H
17	Lipid metabolism	-0.010921916	-0.027117389	0.005273556	0.0283	H
18	Metabolism of other amino acids	-0.012448496	-0.021336983	-0.003560009	0.0155	H

19	Digestive system	-0.01279472	-0.019656478	-0.005932962	0.00276	H
20	Infectious disease: bacterial	-0.01284417	-0.021257725	-0.004430614	0.00157	H
21	Transport and catabolism	-0.013962774	-0.023057502	-0.004868045	0.00867	H
22	Nucleotide metabolism	-0.014991043	-0.047701324	0.017719238	0.0449	H
23	Neurodegenerative disease	-0.015163161	-0.032416402	0.002090081	0.0138	H
24	Translation	-0.017139114	-0.071605921	0.037327694	0.0188	H
25	Metabolism of terpenoids and polyketides	-0.01882226	-0.033441881	-0.004202639	0.0104	H
26	Cell growth and death	-0.01892657	-0.034187593	-0.003665546	0.00802	H
27	Biosynthesis of other secondary metabolites	-0.028432105	-0.045601958	-0.011262252	0.00349	H
28	Xenobiotics biodegradation and metabolism	-0.042297893	-0.098203703	0.013607917	0.0199	H
29	Amino acid metabolism	-0.047662367	-0.09934535	0.004020616	0.00492	H

30	Glycan biosynthesis and metabolism	-0.049242807	-0.104484738	0.005999125	0.0301	H
31	Energy metabolism	-0.053882279	-0.095810756	-0.011953802	0.0197	H
32	Carbohydrate metabolism	-0.097489294	-0.14783775	-0.047140838	0.000572	H

Supplementary Table 17. Analysis of the difference of KEGG pathways between adenoma and intestinal cancer saliva group.

	var	estimate	conf.low	conf.high	p.value	Group
1	Protein families: genetic information processing	0.180414178	0.01722748	0.343600877	0.0171	E
2	sorting and degradation	0.02356043	0.009172358	0.037948501	0.00645	E
3	Folding	0.02356043	0.009172358	0.037948501	0.00645	E
4	Infectious disease: parasitic	-0.002257771	-0.003936046	-0.000579495	0.0207	H
5	Metabolism of other amino acids	-0.009779649	-0.018692483	-0.000866815	0.0358	H
6	Lipid metabolism	-0.017315532	-0.032030255	-0.002600809	0.0155	H

7	Amino acid metabolism	-0.054728305	-0.101096423	-0.008360187	0.026	H
